# Cutaneous Nod2 Expression Regulates the Skin Microbiome and Wound Healing in a Murine Model

**DOI:** 10.1016/j.jid.2017.05.029

**Published:** 2017-11

**Authors:** Helen Williams, Rachel A. Crompton, Helen A. Thomason, Laura Campbell, Gurdeep Singh, Andrew J. McBain, Sheena M. Cruickshank, Matthew J. Hardman

**Affiliations:** 1 Faculty of Biology, Medicine and Health, The University of Manchester, Manchester, UK; 2 School of Life Sciences, University of Hull, Hull, UK

**Keywords:** AMP, antimicrobial peptide, DGGE, density gradient gel electrophoresis, PRR, pattern recognition receptor, qPCR, quantitative real-time PCR, WT, wild type

## Abstract

The skin microbiome exists in dynamic equilibrium with the host, but when the skin is compromised, bacteria can colonize the wound and impair wound healing. Thus, the interplay between normal skin microbial interactions versus pathogenic microbial interactions in wound repair is important. Bacteria are recognized by innate host pattern recognition receptors, and we previously showed an important role for the pattern recognition receptor NOD2 in skin wound repair. NOD2 is implicated in changes in the composition of the intestinal microbiota in Crohn’s disease, but its role on skin microbiota is unknown. *Nod2*-deficient (*Nod2*^*–/–*^) mice had an inherently altered skin microbiome compared with wild-type controls. Furthermore, we found that *Nod2*^*–/–*^ skin microbiome dominated and caused impaired healing, shown in cross-fostering experiments of wild-type pups with *Nod2*^*–/–*^ pups, which then acquired altered cutaneous bacteria and delayed healing. High-throughput sequencing and quantitative real-time PCR showed a significant compositional shift, specifically in the genus *Pseudomonas* in *Nod2*^*–/–*^ mice. To confirm whether *Pseudomonas* species directly impair wound healing, wild-type mice were infected with *Pseudomonas aeruginosa* biofilms and, akin to *Nod2*^*–/–*^ mice, were found to exhibit a significant delay in wound repair. Collectively, these studies show the importance of the microbial communities in skin wound healing outcome.

## Introduction

Skin is colonized by diverse microorganisms, collectively termed the *skin microbiome.* Recent methodological advances in high-throughput sequencing have shown the complexity of microorganisms associated with skin (Grice et al., 2009, NIH HMP Working Group et al., 2009) and have begun to directly implicate a microbial imbalance, a so-called *dysbiosis,* in skin health and disease (Achermann et al., 2014). Our skin is also routinely exposed to potentially pathogenic microorganisms, such as *Staphylococcus aureus* and *Pseudomonas* and *Enterobacter* species (Grice et al., 2009), and has therefore evolved a tightly regulated innate immune response to actively manage the interactions with the skin microbiome.

After injury, it is essential that the skin repairs itself effectively and rapidly. Exposed subcutaneous tissue provides a perfect niche for adventitious pathogens to override the natural microbiome, colonizing the wound (Siddiqui and Bernstein, 2010). Skin cells respond to bacterial invasion via cutaneous pattern recognition receptors (PRRs), including toll-like receptors and the NOD leucine-rich repeat-containing receptors (Kawai and Akira, 2011). PRRs recognize and bind to conserved, pathogen-associated molecular patterns, which ultimately leads to induction of proinflammatory cytokines and secretion of antimicrobial peptides (AMPs) (Kawai and Akira, 2011). NOD2 is an intracellular receptor that recognizes the muramyl dipeptide motif from bacterial peptidoglycans of both Gram-positive and Gram-negative bacteria (Girardin et al., 2003). Mutations in the leucine-rich region of the *NOD2/CARD15* gene are associated with the pathogenesis of several chronic inflammatory diseases of barrier organs including Crohn’s disease (Lesage et al., 2002), asthma (Wong et al., 2015), and Blau syndrome (Kurokawa et al., 2003). Recognition of muramyl dipeptide via NOD2 leads to the activation of the NF-κB pathway, inducing a variety of inflammatory and antibacterial factors. Although a number of studies have highlighted roles for PRRs during cutaneous repair, including members of the toll-like receptor and NOD-like receptor families (Campbell et al., 2013, Dasu et al., 2010, Lai et al., 2009, Lin et al., 2012), the role of PRRs modulating the wound microbiome during repair remains unclear.

Although key studies have provided insight into the regulation of the host-microbiome axis (Grice et al., 2009, Oh et al., 2014), what we now must understand is how cutaneous microorganisms interact with the host and their impact on wound repair. Our previous work showed a previously unreported intrinsic role for murine NOD2 in cutaneous wound healing (Campbell et al., 2013). NOD2 has also been implicated in the regulation of the gut microbiome (Philpott et al., 2014). Given the potential importance of host microbiota/skin interactions during tissue repair, we hypothesized a major link between the NOD2 delayed healing phenotype and the role of NOD2 in cutaneous bacteria modulation. Using a NOD2 null murine model, we show fundamental insights into the role of the innate host response in modulating skin bacteria, with direct effects on tissue repair.

## Results

### *Nod2*-deficient mice have an altered skin microbiome

**Figure 1:**
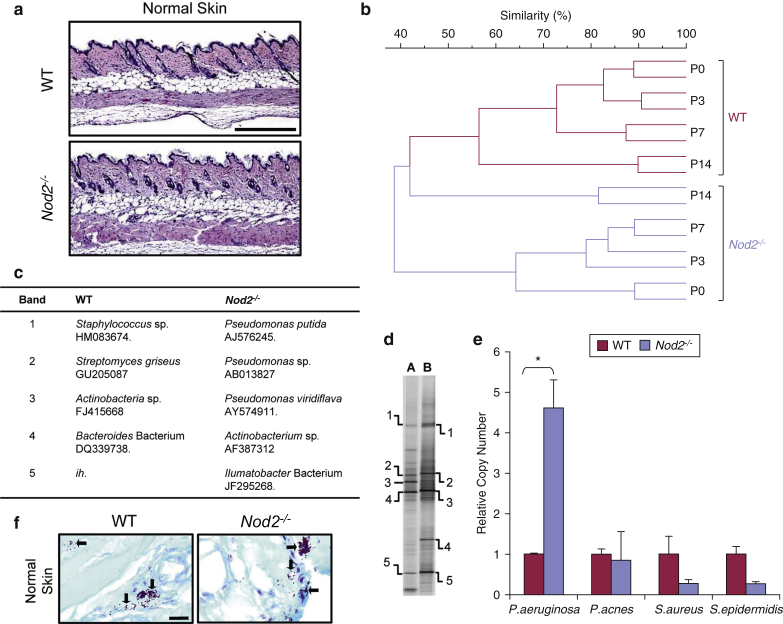
**Skin microbiome dysbiosis in *Nod2*-deficient mice.** (**a**) Representative hematoxylin and eosin-stained sections of normal skin from WT and *Nod2*^*–/–*^ mice show histological equivalence. (**b**) UPGMA dendrogram profiling of WT (green) and *Nod2*^*–/–*^ (purple) skin postnatal development (P0–P14). (**c, d**) DGGE profiles and sequencing of differentially expressed bands showed 40% or less interstrain similarity bacteria between WT and *Nod2*^*–/–*^ mice, and the corresponding bacterial species are illustrated. The table shows putative identity of nearest database match and accession number, and *ih* indicates that the sequence had insufficient homology to enable identification. (**e**) Real-time PCR (16S region) confirmed these species-specific differences between WT and *Nod2*^*–/–*^ normal skin. (**f**) Gram stain of representative histological sections showed that there was a trend toward altered eubacterial abundance in the *Nod2*^*–*^ skin. All data are representative of two independent experiments, with *n* = 2 mice/time point in b, and *n* = 5 mice/group in **c–f**. ^∗^*P* < 0.05. Mean + standard error of the mean. Scale bar in **a** = 200 μm, scale bar in **f** = 20 μm. DGGE, density gradient gel electrophoresis; P, postnatal day; sp., species; UPGMA, unweighted pair group method with arithmetic mean; WT, wild type.

To investigate the role of the PRR Nod2 in the skin, we used the murine *Nod2*^*–/–*^ model. Histologically, the skin of *Nod2*^*–/–*^ mice was comparable to that of wild-type (WT) mice (Figure 1a). However, through density gradient gel electrophoresis (DGGE), we observed major differences in the *Nod2*^*–/–*^ skin microbiome from birth through to adulthood (Figure 1b). 16S rDNA sequencing data of differentially expressed bands indicated enrichment in *Pseudomonas* species (Figure 1c and d), and this was confirmed by quantitative real-time PCR (qPCR), which showed increased relative abundance of *Pseudomonas aeruginosa* in *Nod2*^*–/–*^ skin (Figure 1e) and a trend toward reduced commensal species such as *Staphylococcus epidermidis* (Figure 1e). *P. aeruginosa* is a Gram-negative opportunistic pathogen. Histological Gram staining of skin sections showed no significant difference in the total number of bacteria visualized in the epidermis or dermis. There was, however, a trend toward increased overall numbers of bacterial cells in the dermis of *Nod2*^*–/–*^ skin and a corresponding propensity toward increased abundance of Gram-negative bacteria (see Supplementary Figure S1a–c online).

### Injury exacerbates skin microbiome dysbiosis in *Nod2*-deficient mice

**Figure 2:**
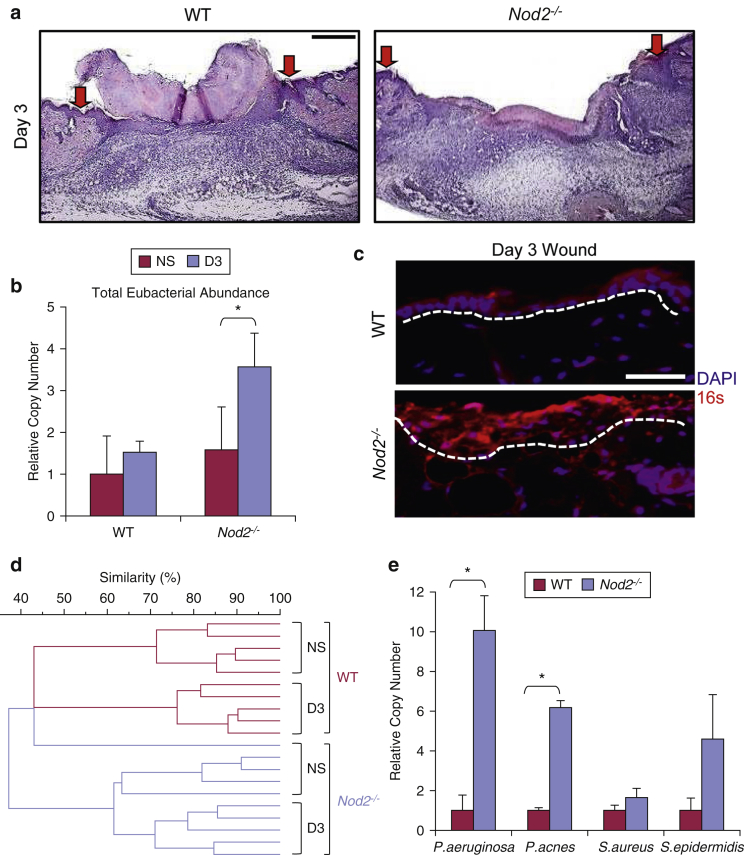
**Wound microbiome dysbiosis in *Nod2*-deficient mice.** (**a**) Representative wounds (day 3) histology showed significantly delayed healing in *Nod2*^*–/–*^ versus WT (arrows denote wound margins). (**b**) *Nod2*^*–/–*^ mice had significantly increased total eubacterial abundance (16S quantititave real-time PCR) in their wounds at postwounding day 3 compared with WT, which was visualized with FISH (**c**) using a total eubacterial FISH probe (red). (**d**) UPGMA dendrogram of wound tissue DGGE fingerprints showed 60% or less interstrain similarity in the *Nod*^*–/–*^ wound microbiome profile versus 40% or less in WT controls. (**e**) Real-time PCR (16S region) confirmed bacterial species-specific differences between WT and *Nod2*^*–/–*^ wounds. All data are representative of two independent experiments with *n* = 5 mice/group (**a–e**). ^∗^*P* < 0.05. Mean + standard error of the mean. Scale bar in **a** = 200 μm, scale bar in **c** = 100 μm. D, day; FISH, fluorescence in situ hybridization; NS, normal skin; UPGMA, unweighted pair group method with arithmetic mean; WT, wild type.

We next addressed the potential contribution of altered skin microbiome to the healing delay observed in *Nod2*^*–/–*^ mice (Campbell et al., 2013) (Figure 2a). Injury increased the total eubacterial abundance in *Nod*^*-–/–*^ but not WT mice (Figure 2b). Fluorescence in situ hybridization confirmed this increased total eubacterial DNA abundance (16S probe) in *Nod2*^*–/–*^ mouse wounds (Figure 2c, quantified in Supplementary Figure S2a–c online). Despite this increase, the bacterial diversity induced by injury was less pronounced in *Nod2*^*–/–*^ than WT mice (≤60% vs. ≤40% respective similarity score between skin and wound) (Figure 2d), which agrees with recent observations from Loesche et al. (2017) that wound microbiota stability is associated with delayed healing. Thus, in the absence of *Nod2,* injury leads to increased relative bacterial abundance, but reduced injury induced changes in bacterial profile. qPCR showed that specific pathogenic species, such as *P. aeruginosa* and *Propionibacterium acnes*, were increased in *Nod2*^*–/–*^ mouse wounds (Figure 2e), which was confirmed by 16S rDNA sequencing (see Supplementary Figure S2d and e). Opportunistic pathogenic species of *Pseudomonas* are linked to chronic inflammation and wound infection (Fazli et al., 2009, Wu et al., 2011) and are thus clear candidates to confer delayed wound healing.

### Antimicrobial peptide expression is altered in *Nod2*-null skin after injury

**Figure 3:**
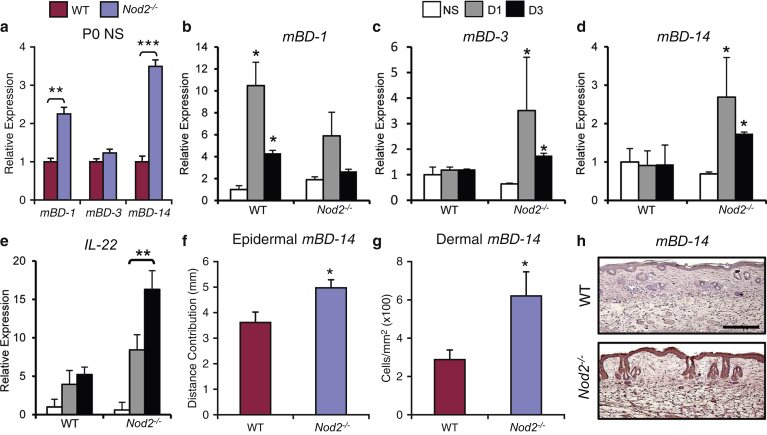
**Injury-induced antimicrobial peptide production is altered in *Nod2*-deficient mice.** (**a**) Cutaneous β-defensins-1 (*mBD-1*) and *-14* were significantly increased from birth (P0) in *Nod2*^*–/–*^ mouse skin versus WT. (**b–e**) In adult *Nod2*^*–/–*^ mice, wounding specifically induced both *mBD-3* and *mBD-14* and *IL-22*. (**f–h**) Immunohistochemical analysis showed increased epidermal and dermal *mBD-14* at 3 days after wounding in *Nod2*^*–/–*^ mice compared with WT. All data are representative of two or three independent experiments with n = 8 mice/group in **a**, and n = 5 mice/group in **b–h**. ^∗∗∗^*P* < 0.001, ^∗∗^*P* < 0.01, ^∗^*P* < 0.05. Mean + standard error of the mean. Scale bar in **h** = 100 μm. D, day; NS, normal skin; P, postnatal day; WT, wild type.

A key component of the antimicrobial host response is the production of AMPs, predominately members of the defensin family (Gallo and Hooper, 2012). Studies in Crohn’s disease patients and *Nod2*-deficient mice showed reduced α-defensin expression in the intestinal mucosa (Eckmann and Karin, 2005, Maeda et al., 2005). Although α-defensins are absent in the skin, specific AMPs including β-defensins are strongly induced in response to cutaneous injury (Ahrens et al., 2011). Unwounded skin of newborn (i.e., with minimal bacterial exposure) *Nod2*^*–/–*^ mice had greater expression of both *mBD-1* and *mBD-14* than matched WT mice (Figure 3a). Adult injury-induced changes in defensin expression also differed between genotypes, with *mBD-1* significantly up-regulated in WT wounds, whereas *Nod2*^*–/–*^ wounds displayed abnormal induction of *mBD-3* and *mBD-14* in response to injury (Figure 3b–d). *IL-22*, a known regulator of mBD-14 expression (Liang et al., 2006), was strongly increased in *Nod2*^*–/–*^ wounds. Finally, we confirmed increased mBD-14 at the protein level in vivo, showing a greater extent of keratinocyte expression and an increased number of mBD-14–positive dermal inflammatory cells in adult wound tissue (Figure 3f–h).

Altered expression of AMPs in the absence of *Nod2* may contribute to an altered microbial community, but equally it may reflect the host response to changes in the composition of the skin microbial community, overall bacterial burden, or the cutaneous location of microbes in the tissue. In experiments analyzing mice born by cesarean, the data showed that cutaneous defensin expression was similar between WT and *Nod2*^*–/–*^ mice (see Supplementary Figure S3 online), suggesting that defensin profiles change in response to microbial challenge. As *Nod2*^*-/-*^ mice had an altered microbiome, an important question was then whether skin dysbiosis was sufficient to alter healing outcome and whether this phenotype could be transferrable.

### Co-housing from birth directly links skin microbiome to healing outcome

**Figure 4:**
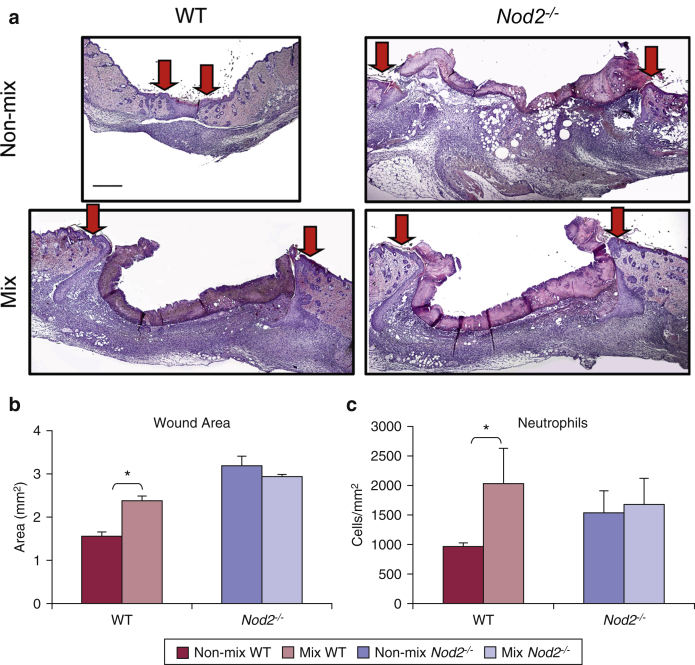
**Co-housing from birth shows that the skin microbiome directly influences healing outcome.** Newborn WT and *Nod2*^*–/–*^ litters were mixed from birth with a *Nod2*^*–/–*^ mother and then wounded in adulthood. (**a**) Representative hematoxylin and eosin-stained sections of excisional wounds (day 5) from WT and *Nod2*^*–/–*^control (non-mix), and co-housed (mix) cages. Arrows denote wound margins. (**b**) Quantification showed significantly delayed healing in WT mice co-housed with *Nod2*^*–/–*^ mice, with (**c**) increased local neutrophil influx. All data are representative of two independent experiments with n = 6 mice/non-mix groups and n = 5 mice/mix group. ^∗^*P* < 0.05. Mean + standard error of the mean. Scale bar in **a** = 200 μm. WT, wild type.

To address causation and to investigate a potential link between bacterial dysbiosis and healing outcome, we mixed newborn WT and *Nod2*^*–/–*^ mice litters from birth with a *Nod2*^*–/–*^ mother. WT mice reared in this mixed environment displayed a clear healing delay with significantly increased wound area (Figure 4a and b) and increased local immune cell recruitment (Figure 4c). The reverse experiment was performed whereby newborn WT and *Nod2*^*–/–*^ litters were co-housed with WT mothers, and although there was no rescue of delayed healing in *Nod2*^*–/–*^ mice, the WT mice had a variable response, with five mice out of eight having delayed healing (see Supplementary Figure S4a and b online) but all showing significantly greater inflammation (see Supplementary Figure S4c), suggesting that the maternal microbiome contribution mediated a partial rescue effect in WT mice.

**Figure 5:**
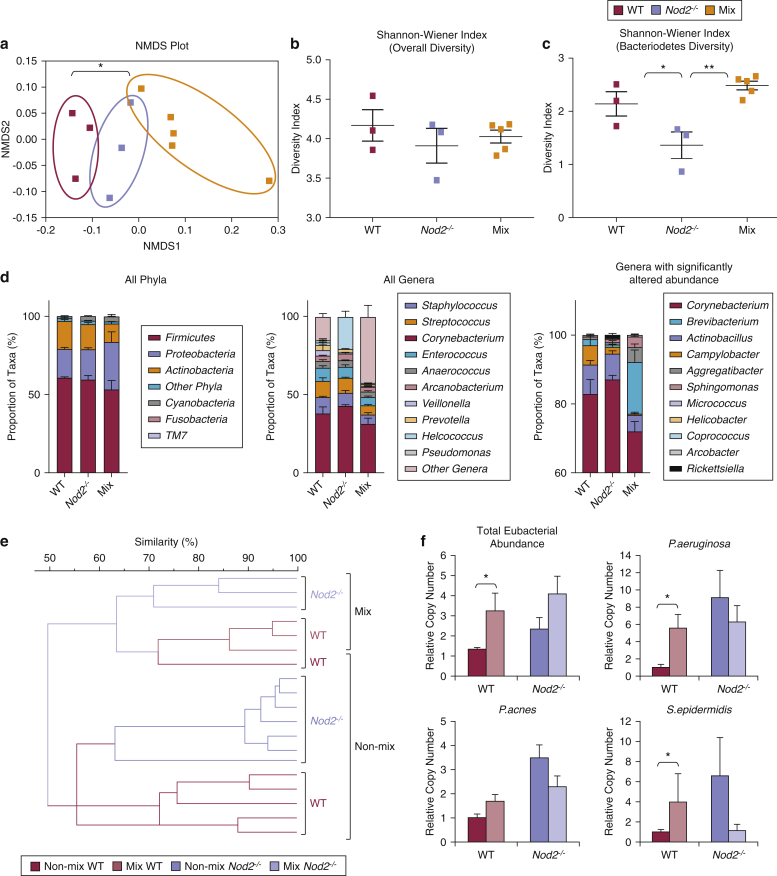
**WT mice co-housed with *Nod2*^*–/–*^ mice from birth acquire pathogenic bacteria.** WT and *Nod2*^*–/–*^ litters were mixed from birth and wounded in adulthood, and their wound microbial communities sequenced. (**a**) NMDS plot showing differences in clustering of microbial communities. Alpha diversity of wound tissue across (**b**) all microbial communities and (**c**) the *Bacteriodetes* phylum was compared using the Shannon-Wiener index. (**d**) Taxonomic classification of the skin microbiome showing proportions of bacteria in each treatment group at the phylum level and genus level and genera that were significantly altered between treatment groups. Individual taxa with abundances too low to visualize clearly and unassigned reads are grouped into the “other” category, which comprises eight additional phyla plus unassigned reads at the phylum level and 219 additional genera plus unassigned reads at the genus level. (**e**) UPGMA dendrogram of WT and *Nod2*^*–/–*^ wound tissue DGGE fingerprints. (**f**) Total wound eubacterial abundance (16S real-time PCR) was significantly increased in WT mice co-housed with *Nod2*^*–/–*^ mice. Mean + standard error of the mean. All data are representative of two independent experiments with n = 3 mice/non-mix groups and n = 5 mice/mix group. ^∗∗^*P* < 0.001, ^∗^*P* < 0.05. NMDS, non-metric multidimensional scaling; UPGMA, unweighted pair group method with arithmetic mean.

Next we analyzed the microbial communities in wound tissue from the co-housing experiment using 16S rRNA Illumina high-throughput sequencing. Nonmetric multidimensional analysis showed statistically significant segregation (*P* < 0.05) based on environment, that is, separately housed mice versus co-housed mice (Figure 5a). There was also a trend toward reduced alpha diversity between each group when compared with WT, as calculated by the Shannon-Wiener index. When focusing on specific skin microbiota at the phylum level, again using the Shannon-Wiener index, there was a significant change in the diversity of *Bacteroidetes* species between environment: separately housed WT versus separately housed *Nod2*^*–/–*^ and separately housed *Nod2*^*–/–*^ versus the co-housed mice (*P* < 0.05 and *P* < 0.01, respectively) (Figure 5c). Furthermore, phylum and genus level taxonomic classification of the wound microbiome is depicted and showed a significantly altered microbial community in separately housed versus co-housed mice, including common skin-associated taxa such as *Corynebacterium* and *Brevibacterium* (Figure 5d). Moreover, the microbial community compositions varied between WT and *Nod2*^*–/–*^ mice, including the genera *Actinobacillus* and *Campylobacter.* The taxonomic information for all mapped reads at the genus level can be found in the Supplementary Materials online (see Supplementary Table S1 online).

Finally, to confirm these differences we also performed DGGE, which showed that mixing of pups resulted in a major shift in the skin microbiome of both genotypes (≤50% similarity versus nonmixed), establishing an intermediate skin bacterial profile (∼65% similarity between genotypes) (Figure 5e). qPCR confirmed that mixed WT wounds acquired increased abundance of specific bacterial species characteristic of *Nod2*^*–/–*^ mice such as *P. aeruginosa*, accompanied by an overall increase in total eubacterial abundance (Figure 5f). Thus, these data provide compelling experimental evidence that skin microbiome directly influences healing outcome.

### Direct administration of *P. aeruginosa* to wild-type mouse wounds significantly delays healing

**Figure 6:**
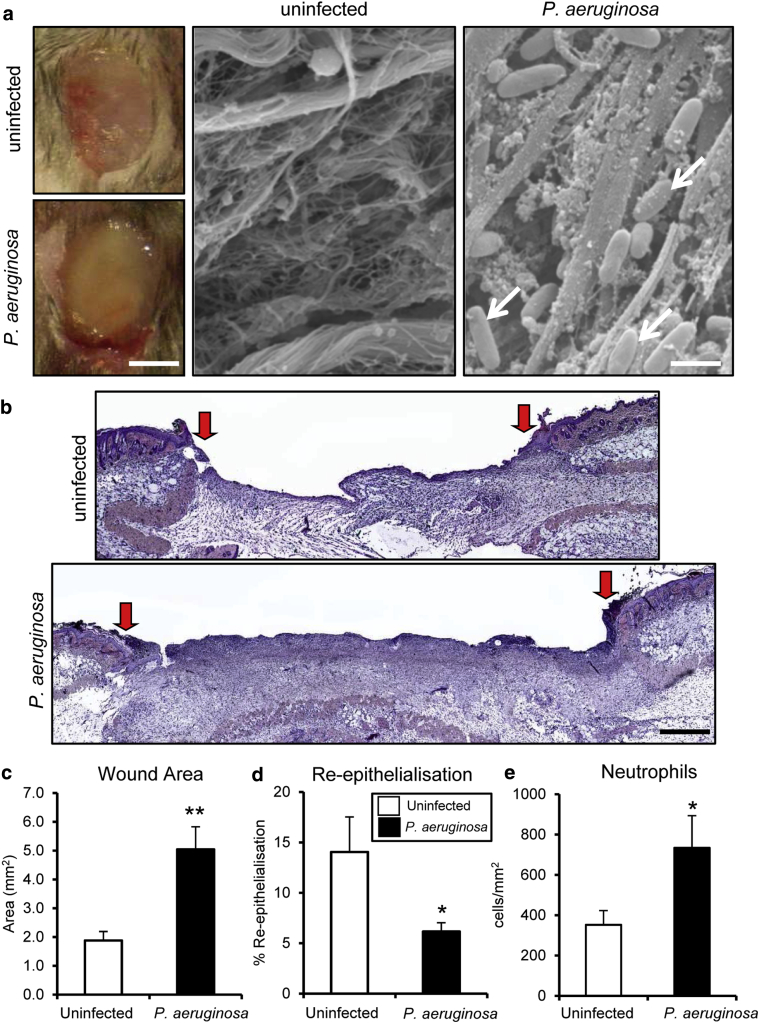
***Pseudomonas aeruginosa* biofilm delays healing in WT mice.** WT mice were inoculated at wounding with a *P. aeruginosa* biofilm. (**a**) Representative images of 3 days postwounding macroscopic and scanning electron microscope images of excisional wounds from normal and *P. aeruginosa* infected mice. Note the characteristic rod-shaped *P. aeruginosa* bacterial cells in the host wound tissue (arrows). (**b**) Representative histological sections illustrate delayed healing in *P. aeruginosa* biofilm wounds. Arrows denote wound margins. (**c–e**) Quantification showed significantly delayed healing in the *P. aeruginosa* group with increased wound area, decreased re-epithelialization, and increased local neutrophil influx at 3 days after wounding. All data are representative of two independent experiments with n = 5 mice/group in **a–e**. ^∗∗^*P* < 0.001, ^∗^*P* < 0.05. Mean + standard error of the mean. Scale bar in **a** (left) = 5 mm, scale bar in **a** (right) = 150 nm, scale bar in b = 200 μm. WT, wild type.

Although we report wide-ranging changes in bacteria in *Nod2*^*–/–*^ mice, a common theme across experiments was increased relative abundance of *Pseudomonas* species. To confirm a direct role for *Pseudomonas* species in wound repair, we treated full-thickness excisional wounds in WT mice with *P. aeruginosa* biofilms and assessed subsequent healing (Figure 6a). Significantly delayed healing was observed after direct application of *P. aeruginosa* to mouse wounds versus nontreated controls (Figure 6b). Treated wounds were larger (Figure 6c), with delayed re-epithelialization (Figure 6d) and increased local inflammation (Figure 6e). These data confirmed that the presence of pathogenic bacteria, similar to wound infection, directly delays murine wound healing and establishes a link to the *Nod2*^*–/–*^ phenotype, where a delay in wound repair is associated with an increased cutaneous presence of the genus *Pseudomonas*.

## Discussion

A wealth of literature has characterized the role of the host response in regulating gut microbiome, with wide-ranging implications for normal physiology and disease (Perez-Chanona et al., 2014, Philpott et al., 2014). By contrast, comparatively few studies have addressed the role of the cutaneous host response-microbiome axis in skin physiology and pathology. We hypothesized that the skin microbiome plays an important role in the cutaneous healing response. Our results show that skin bacterial profiles profoundly influence wound healing outcome. Direct experimental manipulation of the *Nod2* gene leads to bacterial dysbiosis associated with local changes in AMPs and ultimately delays healing. Moreover, when WT mice were co-housed from birth with mice lacking *Nod2,* they acquired an altered microbiome and developed delayed healing. Cutaneous dysbiosis, as shown by eubacterial DNA profiling, 16S high-throughput sequencing, and qPCR, implicated the genus *Pseudomonas* in murine delayed wound repair, and WT mice infected with *P. aeruginosa* biofilms confirmed this. These results suggest that microbial therapy directed at bacterial manipulation of the genus *Pseudomonas,* in addition to other bacterial species previously identified, causes a delay in wound repair, including *S. aureus* and *S. epidermidis* (Schierle et al., 2009), might be an effective strategy to treat wound healing in the future.

A growing body of literature links *NOD2/CARD15* polymorphisms with a dysregulated innate immune response and susceptibility to diseases, including Crohn’s disease (Lesage et al., 2002), Blau syndrome (Kurokawa et al., 2003), early-onset sarcoidosis (Kapral, 1966), and graft-versus-host disease (Hoebe et al., 2005). In the gut, NOD2 has a well-characterized role in host recognition of bacteria and muramyl dipeptide, which is widely expressed by a variety of commensal and pathogenic gut bacteria (Kanneganti et al., 2007, Kawai and Akira, 2010). Studies in patients with Crohn’s disease and *Nod2*-deficient mice showed that intestinal changes in bacterial composition are associated with altered α-defensin expression in the intestinal mucosa (Philpott et al., 2014). α-Defensins are not expressed in skin; however, the cutaneous effects of NOD2 are associated with altered β-defensins, yet the exact role these AMPs are playing in our *Nod2-*null mice remain to be elucidated. Changes in skin β-defensins have previously been linked to skin infection (e.g., *Staphylococcus aureus*) (Zanger et al., 2010) and skin disease (e.g., atopic dermatitis) (Ong et al., 2002). Thus, a picture is emerging across multiple epithelial tissues whereby a loss of NOD2-mediated surveillance activity inhibits local host responses to pathogenic challenge, resulting in aberrant inflammation and bacterial dysbiosis.

All wounds will be rapidly colonized by resident bacteria, but only some wounds will become “infected.” Considerable recent interest has been focused on the potential ability of these colonizing bacteria to form and exist as highly-AMP resistant polymicrobial biofilms (Malone et al., 2017). A number of bacterial genera/species, such as *Streptococcus*, *Enterococcus, S. aureus,* and *P. aeruginosa,* have already been linked to infected chronic wounds (Bjarnsholt, 2013, James et al., 2016, Zhao et al., 2014). However, the clinical diagnosis for wound infection (in humans) is based on the basic criteria of heat, odor and appearance. In this study we show that similarly appearing murine acute (noninfected) wounds display differences in wound microbiota profile that clearly influence healing outcome.

Arguably the most important finding in this study comes from the newborn mouse co-housing experiments, where passive transfer of skin bacteria from *Nod2*-null to WT mice conferred a “NOD2-like” delayed healing phenotype. The concept of transferring signature bacterial profiles to closely related individuals has now been established clinically. For example, unaffected relatives of Crohn’s disease patients reportedly share some features of the disease-associated microbiome composition (Joossens et al., 2011). Fecal transplant, also referred to as *gut microbiome transplant,* a procedure in which fecal bacteria from a healthy donor are transplanted into a patient, has shown promise in the treatment of Crohn’s disease and ulcerative colitis (Young, 2016). Similarly, cross-strain murine relocation/uterine implantation studies showed that environmental influences dominate the gastrointestinal tract microbiome (Friswell et al., 2010). Our data strongly suggest that the cutaneous microbiome is also highly susceptible to environmental influences, with clear functional consequences. Finally, our data suggest a potential therapeutic opportunity for the treatment of cutaneous dysbiosis in relation to wound repair via microbial manipulation of the skin microbiome. Indeed, mounting research suggests the profound benefits of probiotic supplementation for gut microbiota in health and disease (Gareau et al., 2010, Rolfe, 2000). These may now be extended to other epithelia, including the skin (Mohammedsaeed et al., 2014).

## Materials and Methods

### Animals and wounding

All animal studies were performed in accordance with UK Home Office Regulations. All mice used in this study were bred in the same room under the same conditions at the University of Manchester’s Biological Services Facility, where they have been housed for 10 or more generations. Mice were housed in isolator cages with ad libitum food and water. The room was maintained at a constant temperature of 21 °C, with 45–65% humidity on a 12-hour light-dark cycle. *Nod2-*null mice (C57BL/6J background) were bred from homozygous matings and have been described previously (Campbell et al., 2013). WT (C57BL/6J) mice were bred from WT × WT matings onsite to generate controls for experiments. Eight-week-old female mice were anesthetized and wounded following our established protocol (Ansell et al., 2014). Briefly, two equidistant 6-mm full-thickness excisional wounds were made through both skin and panniculus carnosus muscle and left to heal by secondary intention. For co-housing experiments, mice were marked by tattooing, and then 2 or 3 tattooed pups (postnatal day 0) of one genotype were placed in the same cage with 2 or 3 tattooed pups (postnatal day 0) of the other genotype and fostered onto WT or *Nod2*^*–/–*^ mothers for at least 5 weeks before separation (for weaning). After weaning, only mice of the same sex were housed together before wounding at 6 weeks.

### *P. aeruginosa*-infected mouse model

An overnight broth culture of *P. aeruginosa* (National Collection of Type Cultures 10781) was diluted to turbidity equivalent to 0.5 McFarland standard (optical density = 0.132 at 600 nm) in Mueller-Hinton broth (Oxoid, Hampshire, UK). A total of 50 μl of the diluted culture was applied to 6-mm–diameter sterile 0.2-μm filter membranes (Merck Millipore, Hertfordshire, UK) placed on Mueller-Hinton agar plates. These were then incubated at 37 °C for 72 hours, with transfer to a new agar plate every 24 hours. The resultant biofilms were applied to 6-mm excisional wounds and covered with a nonwoven Sawabond 4383 dressing (Sandler, Schwarzenbach/Saale, Germany).

### Collection of murine tissue and contralateral skin swabs

Excisional wounds were harvested at 1, 3, and 5 days after wounding and bisected (laterally at the midpoint), with one half placed on dry ice for DGGE analysis or fixed in formalin for histology and the remaining half snap-frozen in liquid nitrogen and stored at –80 °C. Skin swabs from an area of intact contralateral skin were also collected using sterile Dual Amies transport swabs (Duo Transwab; MWE, Wiltshire, UK) and inoculated into 1 ml of transport medium and processed within 3 hours of collection.

### DNA extraction from tissue samples and manipulation

All biological specimens were incubated in enzymatic lysis buffer (20 mmol/L Tris at pH 8.0, 0.2 mmol/L EDTA, 1.2% triton X-100) and lysozyme (20 mg/ml) for 30 minutes at 37 °C. DNA was extracted using a Qiagen DNeasy blood and tissue kit (Qiagen., West Sussex, UK) in accordance with the manufacturer’s instructions but with the added step of using 0.1-mm sterile zirconia/silica beads (BioSpec, Bartlesville, OK) to homogenize the samples.

### PCR amplification and purification

The V3 variable region of the 16S rRNA gene was amplified from purified DNA by PCR using GC-rich eubacterium-specific primers P3_GC-341F and 518R (see Supplementary Table S2 online) as previously described (Walter et al., 2000) using a PTC-100 DNA Engine thermal cycler (Bio-Rad MJ Research, Hertfordshire, UK). Samples were purified using a Qiagen MinElute purification kit (Qiagen.) in accordance with manufacturer’s instructions.

### DGGE

Polyacrylamide electrophoresis was performed using the D-CODE Universal Mutation Detection System (Bio-Rad, Hertfordshire, UK) according to the manufacturer’s instructions for perpendicular DGGE. Denaturing gradient gels of 10% (weight/volume) acrylamide-bisacrylamide (37:1:5) were made (Fisher Scientific, Loughborough, UK) containing a 30–70% linear gradient of denaturants (urea and formamide), increasing in the direction of electrophoresis as described previously (Walter et al., 2000). DGGE gel images were aligned and analyzed with BioNumerics software version 4.6 (Applied Maths, Sint-Martens-Latem, Belgium) in a multistep procedure following the manufacturer’s instructions. After normalizations of the gels, individual bands in each lane of the gel were detected automatically, allowing matching profiles to be generated and used to produce an unweighted pair group method with arithmetic mean dendrogram.

### Excision and sequencing of DGGE bands

Selected bands were sterilely excised from the gel under UV illumination in 20 μl Nanopure H_2_O (Millipore, Hertfordshire, UK) in nuclease-free tubes. PCR products were purified using QIAquick PCR purification kit (Qiagen.) and re-amplified using the reverse 518R primer. Sequencing was performed using BigDye terminator sequencing on an ABI 3730 genetic analyzer (Applied Biosystems by Life Technologies, Paisley, UK) for Sanger sequencing. Sequences obtained were compared with those in the European Molecular Biology Laboratory nucleotide sequence database using Basic Local Alignment Search Tool (BLAST) (National Library of Medicine, Bethesda, MD, USA) searches to identify closely related gene sequences.

### 16S rRNA gene sequencing analysis

16S amplicon sequencing targeting the V3 and V4 variable region of the 16S rRNA gene (see Supplementary Table S2) was performed on the Illumina MiSeq platform (Illumina Inc, Cambridge, UK). The raw amplicon data were further processed using quantitative insights into microbial ecology (i.e., QIIME) version 1.9.0 (Caporaso et al., 2010) and R version 3.3.1 (R Core Team, 2016). The non-metric multidimensional scaling plot and the Shannon-Weiner index were created using the isoMDS function in the MASS package (Venables and Ripley, 2002) in R, and the statistical analysis was performed using the Adonis function in the vegan package (Okansen et al., 2016) in R.

### Hucker-Twort Gram stain

The Hucker-Twort Gram stain was used to distinguish Gram-positive and Gram-negative bacteria in formalin-fixed tissue. Tissue was flooded with crystal violet stain for 3 minutes and rinsed with running H_2_O. Gram’s iodine was added for 3 minutes and washed with H_2_O. After differentiation in preheated acetic alcohol at 56 °C, tissue was immersed with Twort’s stain for 5 minutes and washed with H_2_O. Slides were rinsed in alcohol, cleared in xylene, and mounted with DPX mountant (Sigma-Aldrich, Dorset, UK); the slides were imaged using a 3D Histech Pannoramic 250 Flash Slide Scanner (3D Histech, Budapest, Hungary).

### qPCR

Bacterial DNA and/or total RNA was isolated from frozen skin or wound tissue as previously described or by homogenizing in Trizol reagent using the Purelink RNA kit (Invitrogen by Life Technologies., Paisley, UK) according to the manufacturer’s instructions. cDNA was transcribed from 1 μg of RNA (Promega RT Kit, Hampshire, UK) and AMVreverse transcriptase (Roche, West Sussex, UK), and qPCR performed using the SYBR Green I core kit (Eurogentec, Hampshire, UK) and an Opticon quantitative PCR thermal cycler (BioRad, Hertfordshire, UK). The primer sequences for real-time qPCR are listed in Supplementary Table S2.

### Histology and immunohistochemistry

Histological sections were prepared from normal skin and wound tissue fixed in 10% buffered formalin saline and embedded in paraffin. 5-μmol/L sections were stained with hematoxylin and eosin or subjected to immunohistochemical analysis using the following antibodies: rat anti-neutrophil polyclonal (Fisher Scientific) and chicken anti-BD-14 polyclonal (a generous gift from Thomas Hehlgans, University of Regensburg, Regensburg, Germany). Primary antibody was detected using the appropriate biotinylated secondary antibody followed by ABC-peroxidase reagent (Vector Laboratories, Peterbourgh, UK) with NovaRed substrate and counterstaining with hematoxylin. Images were captured using an Eclipse E600 microscope (Nikon, Surrey, UK) and a SPOT camera (Image Solutions., Preston, UK). Total cell numbers, bacterial counts, granulation tissue wound area, and re-epithelialization were quantified using Image Pro Plus software (Media Cybernetics, Buckinghamshire, UK).

### Fluorescence in situ hybridization

The deparaffinized tissue sections were systematically analyzed by fluorescence in situ hybridization using peptide nucleic acid probes. A mixture of a CY3-labelled universal bacterium peptide nucleic acid probe in hybridization solution (AdvanDx., Woburn, MA) was added to each section and hybridized in a peptide nucleic acid fluorescence in situ hybridization workstation at 55 °C for 90 minutes. Slides were washed for 30 minutes at 55 °C in wash solution (AdvanDx.), mounted in DAPI-containing mountant, and stored in the dark at –20 °C. Slides were visualized using a DMLB 100s Leica Microsystems microscope attached to a Leica Microsystems fluorescence system (Lecia, Milton Keynes, UK). Images were captured using a RS Phototmetrics Coolsnap camera (Photometrics, Tucson, AZ) and overlaid using Adobe Photoshop Elements version 6.5 (Adobe, San Jose, CA).

### Electron microscopy

Samples were processed as previously described (Kimura et al., 2007), with the exception that 4% paraformaldehyde and 2 mmol/L CaCl_2_ were used in the primary fixative and 2% OsO_4_ in the secondary fixative. Images were acquired using the Orius CDD SC1000 camera (Gatan, Oxon, UK).

### Statistical analysis

All data are presented as mean + standard error of the mean. Normal distribution and statistical comparisons between groups were determined using Shapiro-Wilk test, Student *t* test (two tailed), or two-way analysis of variance with Bonferroni posttest where appropriate using GraphPad Prism 7 version 7.01 (GraphPad Software, La Jolla, CA) as indicated in the figure legends. For all statistical tests, the variance between each group was determined, and probability values of *P* less than 0.05 were considered statistically significant.

## Conflict of Interest

The authors state no conflict of interest.
