# Supplementary material for: Cutaneous Nod2 Expression Regulates the Skin Microbiome and Wound Healing in a Murine Model
Source: J Invest Dermatol. 2017 Nov;137(11):2427–36. doi: 10.1016/j.jid.2017.05.029 (PMC5646944; doi:10.1016/j.jid.2017.05.029)
Supplement: Supplementary Figures S1–S4 and Supplementary Tables S1 and S2 [file mmc1.pdf]

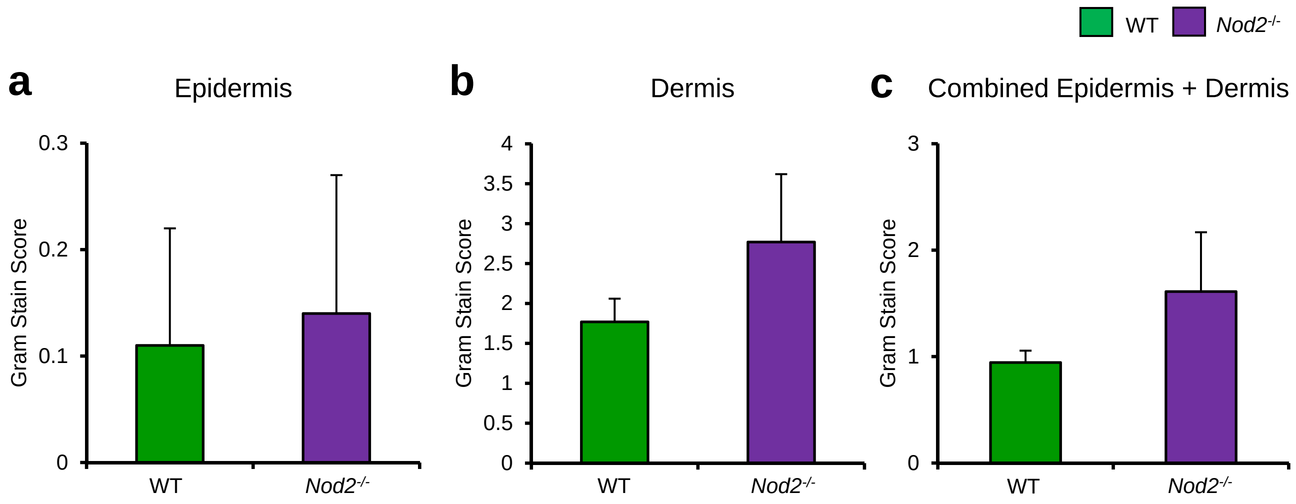

<u>Supplementary Figure S1. Gram-stain quantification</u>. Quantification of Gram-staining showed there was a no significant difference in total eubacterial burden in both WT vs. *Nod2*$^{-/-}$ normal skin (a) epidermis, (b) dermis and (c) combined epidermal and dermal scores. There was, however, a trend towards altered eubacterial abundance in the dermis (b) of *Nod2*$^{-/-}$ normal skin. All data are representative of two independent experiments, with *n* = 5 mice/group. Mean + standard error of the mean. WT, wild type.

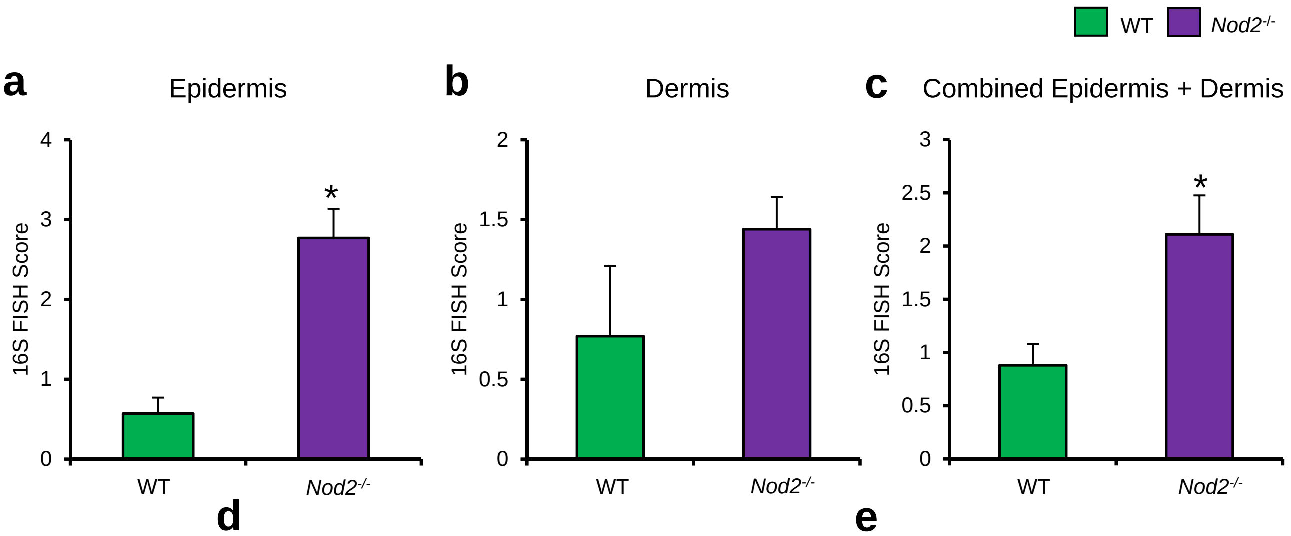

d

Band	WT	Nod2$^{-/-}$
1	*Staphylococcus* aureus. YP040448	Uncultured actinbacterium. GCP1
2	*Staphylococcus* epidermidis. D83363	*Pseudomonas* sp. AF320988
3	*Pseudomonas* sp. AB013827.	*Pseudomonas* fragi. AHZY01000001.
4	*Actinobacteria* sp. clK1_14b.	*Arenibacter* certessi. CCUG48006T.

Putative identity of nearest database match and accession number:
ih, sequence insufficient homology to enable identification;

e

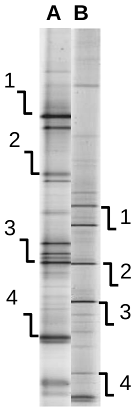

Supplementary Figure S2. Wound microbiome dysbiosis in *Nod2*-deficient mice. (a-c) Quantification of 16S FISH confirmed a significant increase in total eubacterial abundance in *Nod2*$^{-/-}$ day 3 wounds. (d-e) 16S rDNA sequencing of differentially expressed bands from the DGGE fingerprints revealed the bacterial species in wounded WT and *Nod2*$^{-/-}$ mice. All data are representative of two independent experiments, with n = 5 mice/group. * P<0.05. Mean + standard error of the mean. FISH, fluorescence in situ hybridization; sp., species; WT, wild type.

a

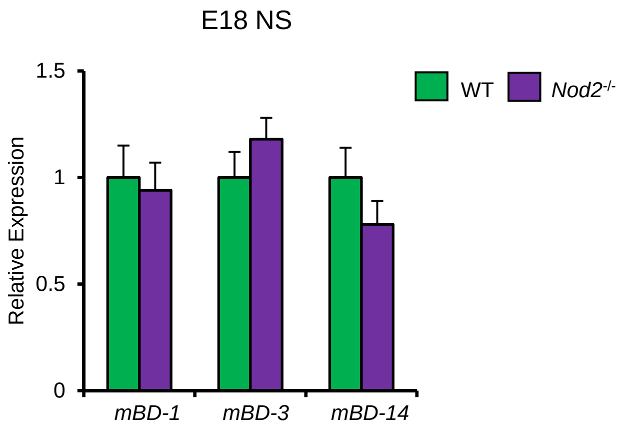

<u>Supplementary Figure S3. Absence of altered antimicrobial peptide production in *Nod2*-deficient mice obtained via caesarean birth.</u> (a) There was no significant difference in cutaneous *mBD-1*, *-3* or *-14* in *Nod2*$^{-/-}$ mouse skin versus WT from mice born via caesarean section. All data are representative of two independent experiments with *n* = 8-10 mice/group. Mean + standard error of the mean. E, embryonic day; WT, wild type.

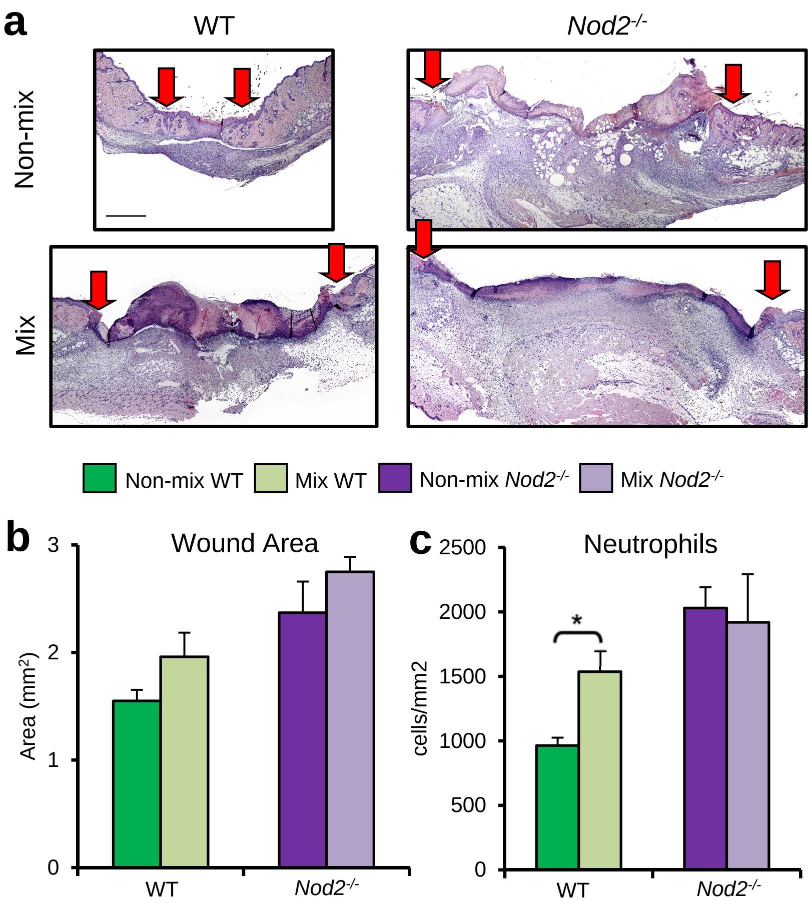

Supplementary Figure S4. Co-housing from birth with a WT mother shows the skin microbiome directly influences healing outcome. Newborn WT and *Nod2*$^{-/-}$ litters were mixed from birth with a WT mother, and then wounded in adulthood. (a) Representative H+E stained sections of excisional wounds (day 5) from WT and *Nod2*$^{-/-}$ control (non-mix), and co-housed (mix) cages (arrows denote wound margins). (b) Quantification showed significantly delayed healing in *Nod2*$^{-/-}$ mice co-housed with WT mice, with (c) increased local neutrophil influx. All data are representative of two independent experiments with n = 6 mice/non-mix groups and n = 5 mice/mix group. * P<0.05. Mean + stand error of the mean. Scale bar = 250 µM (a). WT, wild type.

Supplementary Table S1. Average proportion of genera for all mapped reads from the 16S Illumina high-throughput sequencing data, including probability values as determined by one-way ANOVA.

	WT	*Nod2*$^{-/-}$	Mix	P value
k__Bacteria.p__Bacteroidetes.c__Flavobacteriia.o__Flavobacteriales.f__.Weeksellaceae..g__	0.00314	0	0.00018	0.00019
k__Bacteria.p__GN02.c__BD1.5.o__.f__.g__	0.00011	0.00162	0	0.00354
k__Bacteria.p__Actinobacteria.c__Actinobacteria.o__Actinomycetales.f__Micrococcaceae.g__Micrococcus	4.8E-05	0.00058	0.00176	0.00457
k__Bacteria.p__Bacteroidetes.c__Flavobacteriia.o__Flavobacteriales.f__Flavobacteriaceae.g__	0.00205	0.00673	0.00203	0.00998
k__Bacteria.p__Proteobacteria.c__Gammaproteobacteria.o__Legionellales.f__Coxiellaceae.g__Rickettsiella	0	0.00098	0	0.01268
k__Bacteria.p__OD1.c__ZB2.o__.f__.g__	0.00041	0.00294	0.00026	0.01494
k__Bacteria.p__Firmicutes.c__Clostridia.o__Clostridiales.f__Lachnospiraceae.g__Coprococcus	0	0	0.00032	0.01507
k__Bacteria.p__Proteobacteria.c__Epsilonproteobacteria.o__Campylobacterales.f__Campylobacteraceae.g__Campylobacter	0.00143	0	0	0.01603
k__Bacteria.p__Actinobacteria.c__Actinobacteria.o__Actinomycetales.f__Brevibacteriaceae.g__Brevibacterium	0.0082	0.00633	0.0035	0.0169
k__Bacteria.p__TM7.c__SC3.o__.f__.g__	0	0.0006	0	0.01718
k__Bacteria.p__Proteobacteria.c__Gammaproteobacteria.o__Pasteurellales.f__Pasteurellaceae.g__Actinobacillus	0.00559	0	0.00018	0.01868
k__Bacteria.p__Proteobacteria.c__Gammaproteobacteria.o__Enterobacteriales.f__Enterobacteriaceae.g__	0.116	0.12887	0.09599	0.02616
k__Bacteria.p__Proteobacteria.c__Alphaproteobacteria.o__Sphingomonadales.f__Sphingomonadaceae.g__Sphingomonas	0.00049	0.0006	0.00259	0.02692
k__Bacteria.p__Proteobacteria.c__Gammaproteobacteria.o__Pasteurellales.f__Pasteurellaceae.g__Aggregatibacter	0.00057	0.0017	0.01141	0.02792
k__Bacteria.p__Firmicutes.c__Bacilli.o__Lactobacillales.f__Leuconostocaceae.g__	0.00275	0.00105	0.00049	0.02851
k__Bacteria.p__Proteobacteria.c__Epsilonproteobacteria.o__Campylobacterales.f__Campylobacteraceae.g__Arcobacter	0	0.00077	0	0.03126
k__Bacteria.p__Actinobacteria.c__Actinobacteria.o__Actinomycetales.f__Corynebacteriaceae.g__Corynebacterium	0.08297	0.07383	0.04718	0.0387
k__Bacteria.p__Proteobacteria.c__Epsilonproteobacteria.o__Campylobacterales.f__Helicobacteraceae.g__Helicobacter	9.57E-06	0	4E-05	0.04988
k__Bacteria.p__Proteobacteria.c__Betaproteobacteria.o__Burkholderiales.f__Oxalobacteraceae.g__	9.6E-05	0	0.00151	0.05335
k__Bacteria.p__Proteobacteria.c__Epsilonproteobacteria.o__Campylobacterales.f__Campylobacteraceae.g__Sulfurospirillum	0	0	0.00084	0.05345
k__Bacteria.p__Firmicutes.c__Clostridia.o__Clostridiales.f__Ruminococcaceae.g__	0	0.0016	0.0033	0.05784
k__Bacteria.p__Firmicutes.c__Bacilli.o__Bacillales.f__Planococcaceae.g__Paenisporosarcina	0	0	0.00029	0.05944
k__Bacteria.p__Firmicutes.c__Bacilli.o__Bacillales.f__.Exiguobacteraceae..g__Exiguobacterium	0.00013	4.8E-05	0.03356	0.06115
k__Bacteria.p__Proteobacteria.c__Epsilonproteobacteria.o__Campylobacterales.f__Helicobacteraceae.Other	0.00022	0.00087	0.0029	0.063
k__Bacteria.p__Firmicutes.c__Clostridia.o__Clostridiales.f__Veillonellaceae.g__	9.6E-05	0	0.00108	0.07044
k__Bacteria.p__Proteobacteria.c__Alphaproteobacteria.o__Rhizobiales.f__Phyllobacteriaceae.Other	0.00948	0.00769	0.00511	0.08109
k__Bacteria.p__Proteobacteria.c__Deltaproteobacteria.o__Bdellovibrionales.f__Bacteriovoracaceae.g__	0	0.00023	0	0.08894
k__Bacteria.p__Proteobacteria.c__Betaproteobacteria.o__Burkholderiales.f__Burkholderiaceae.g__Burkholderia	5.74E-05	0.00116	0.00033	0.09309
k__Bacteria.p__Firmicutes.c__Bacilli.o__Lactobacillales.f__Leuconostocaceae.g__Leuconostoc	0	0.0006	0.00221	0.10767
k__Bacteria.p__Actinobacteria.c__Coriobacteriia.o__Coriobacteriales.f__Coriobacteriaceae.g__Atopobium	0.00337	0.00104	0.00204	0.10879
k__Bacteria.p__Firmicutes.c__Bacilli.o__Lactobacillales.f__Streptococcaceae.g__Lactococcus	0	0	0.0005	0.11566
k__Bacteria.p__Bacteroidetes.c__Bacteroidia.o__Bacteroidales.f__Porphyromonadaceae.g__Parabacteroides	0	3.8E-05	0.00076	0.11989
Unassigned.Other.Other.Other.Other.Other	0.0148	0.01508	0.00966	0.12002
k__Bacteria.p__Actinobacteria.c__Actinobacteria.o__Actinomycetales.f__Micrococcaceae.Other	0.00057	0.00282	0.00121	0.12411
k__Bacteria.p__Firmicutes.c__Bacilli.o__Lactobacillales.f__Streptococcaceae.g__Streptococcus	0.08336	0.0631	0.04731	0.13493
k__Bacteria.p__Firmicutes.c__Bacilli.o__Lactobacillales.f__Enterococcaceae.g__Enterococcus	0.06768	0.05184	0.04181	0.15836
k__Bacteria.p__Proteobacteria.c__Epsilonproteobacteria.o__Campylobacterales.f__Helicobacteraceae.g__	0	0.00113	6.89E-05	0.17481
k__Bacteria.p__Firmicutes.c__Clostridia.o__Clostridiales.f__Veillonellaceae.g__vadinHB04	0	0	0.0003	0.17855
k__Bacteria.p__Firmicutes.c__Bacilli.o__Bacillales.f__Planococcaceae.g__Lysinibacillus	0	0.00055	0	0.17906
k__Bacteria.p__Proteobacteria.c__Gammaproteobacteria.o__Enterobacteriales.f__Enterobacteriaceae.g__Proteus	0.00122	0.00269	0.00085	0.18763
k__Bacteria.p__Firmicutes.c__Bacilli.o__Bacillales.f__Staphylococcaceae.g__Staphylococcus	0.30266	0.32254	0.24689	0.18925
k__Bacteria.p__Firmicutes.c__Clostridia.o__Clostridiales.f__Dehalobacteriaceae.g__Dehalobacterium	0	0.00019	0	0.19852
k__Bacteria.p__Proteobacteria.c__Gammaproteobacteria.o__Pseudomonadales.f__Pseudomonadaceae.g__	2.9E-05	0.00057	0.00557	0.19956
k__Bacteria.p__Firmicutes.c__Bacilli.o__Bacillales.f__Planococcaceae.Other	0	0.00011	0.00015	0.19993
k__Bacteria.p__Firmicutes.c__Bacilli.o__Lactobacillales.Other.Other	0.00171	0.00284	0.0011	0.2107
k__Bacteria.p__Verrucomicrobia.c__Verrucomicrobiae.o__Verrucomicrobiales.f__Verrucomicrobiaceae.g__Akkermansia	0	0.00088	0.00014	0.21867
k__Bacteria.p__Proteobacteria.c__Gammaproteobacteria.o__Enterobacteriales.f__Enterobacteriaceae.g__Citrobacter	0	0.00104	0.00281	0.22641
k__Bacteria.p__Firmicutes.c__Bacilli.o__Lactobacillales.f__Carnobacteriaceae.Other	9.57E-06	0	2.9E-05	0.23156
k__Bacteria.p__Actinobacteria.c__Actinobacteria.o__Actinomycetales.f__Mycobacteriaceae.g__Mycobacterium	0	0	0.00049	0.23489
k__Bacteria.p__Fusobacteria.c__Fusobacteriia.o__Fusobacteriales.f__Leptotrichiaceae.g__	0.00093	0	0	0.24174
k__Bacteria.p__Proteobacteria.c__Betaproteobacteria.o__Burkholderiales.f__Oxalobacteraceae.Other	0	0	0.00165	0.24274
k__Bacteria.p__Proteobacteria.c__Betaproteobacteria.o__Burkholderiales.f__Comamonadaceae.g__Tepidimonas	0	0	0.00025	0.25053
k__Bacteria.p__Proteobacteria.c__Gammaproteobacteria.o__Pseudomonadales.f__Moraxellaceae.g__Acinetobacter	0.00331	0.00874	0.12338	0.25066
k__Bacteria.p__Actinobacteria.c__Actinobacteria.o__Actinomycetales.f__.g__	0.02757	0.0244	0.01827	0.26326
k__Bacteria.p__Actinobacteria.c__Actinobacteria.o__Actinomycetales.f__Dermabacteraceae.g__Dermabacter	0.00097	0.00123	0.00026	0.26966
k__Bacteria.p__Firmicutes.c__Bacilli.o__Lactobacillales.f__Carnobacteriaceae.g__Granulicatella	0.00174	2.9E-05	0.00029	0.27766
k__Bacteria.p__Proteobacteria.c__Betaproteobacteria.o__Neisseriales.f__Neisseriaceae.g__	0.00055	0	0.00015	0.27905
k__Bacteria.Other.Other.Other.Other.Other	0	0	1.1E-05	0.2892
k__Bacteria.p__Actinobacteria.c__Actinobacteria.o__Actinomycetales.f__Dermabacteraceae.Other	0	9.57E-06	0	0.2892
k__Bacteria.p__Actinobacteria.c__Actinobacteria.o__Actinomycetales.f__Microbacteriaceae.Other	0	9.57E-06	2.3E-05	0.2892
k__Bacteria.p__Actinobacteria.c__Actinobacteria.o__Actinomycetales.f__Microbacteriaceae.g__Leucobacter	0	0.00031	0	0.2892
k__Bacteria.p__Actinobacteria.c__Coriobacteriia.o__Coriobacteriales.f__Coriobacteriaceae.g__	0.0002	0	0	0.2892
k__Bacteria.p__Armatimonadetes.c__Chthonomonadetes.o__Chthonomonadales.f__Chthonomonadaceae.g__Chthonomonas	0.00054	0	0	0.2892
k__Bacteria.p__Armatimonadetes.c__.Fimbriimonadia..o__.Fimbriimonadales..f__.Fimbriimonadaceae..g__Fimbriimonas	0	0.00019	0	0.2892
k__Bacteria.p__Bacteroidetes.c__Bacteroidia.o__Bacteroidales.f__.Paraprevotellaceae..g__.Prevotella.	0.00563	0	0	0.2892
k__Bacteria.p__Bacteroidetes.c__Flavobacteriia.o__Flavobacteriales.f__Cryomorphaceae.g__Fluviicola	0	0.00026	0	0.2892
k__Bacteria.p__Bacteroidetes.c__Flavobacteriia.o__Flavobacteriales.f__.Weeksellaceae..g__Wautersiella	0	9.57E-06	0	0.2892
k__Bacteria.p__Bacteroidetes.c__Sphingobacteriia.o__Sphingobacteriales.f__.g__	6.7E-05	0	0	0.2892
k__Bacteria.p__Bacteroidetes.c__.Saprospirae..o__.Saprospirales..f__Chitinophagaceae.g__Sediminibacterium	1.91E-05	0	0	0.2892
k__Bacteria.p__Bacteroidetes.c__.Saprospirae..o__.Saprospirales..f__Saprospiraceae.g__	0	0.0002	0	0.2892
k__Bacteria.p__Cyanobacteria.c__Chloroplast.o__Cryptophyta.f__.g__	0	0.00013	0	0.2892
k__Bacteria.p__Cyanobacteria.c__Chloroplast.o__Stramenopiles.f__.g__	4.8E-05	0	0	0.2892
k__Bacteria.p__Cyanobacteria.c__Synechococcophycideae.o__Synechococcales.f__Synechococcaceae.g__Synechococcus	0	0.00059	0	0.2892
k__Bacteria.p__Firmicutes.Other.Other.Other.Other	0	0	1.1E-05	0.2892
k__Bacteria.p__Firmicutes.c__Bacilli.o__Bacillales.f__Bacillaceae.g__Anoxybacillus	1.91E-05	0	0	0.2892
k__Bacteria.p__Firmicutes.c__Bacilli.o__Bacillales.f__Bacillaceae.g__Geobacillus	0	0.00023	0	0.2892
k__Bacteria.p__Firmicutes.c__Bacilli.o__Bacillales.f__Paenibacillaceae.g__Paenibacillus	0	4.8E-05	0	0.2892
k__Bacteria.p__Firmicutes.c__Bacilli.o__Bacillales.f__Staphylococcaceae.g__Salinicoccus	0	9.57E-06	0	0.2892
k__Bacteria.p__Firmicutes.c__Bacilli.o__Bacillales.f__Thermoactinomycetaceae.g__	1.91E-05	0	0	0.2892
k__Bacteria.p__Firmicutes.c__Bacilli.o__Lactobacillales.f__Carnobacteriaceae.g__Trichococcus	0	0	1.1E-05	0.2892
k__Bacteria.p__Firmicutes.c__Bacilli.o__Lactobacillales.f__Leuconostocaceae.Other	0	9.57E-06	0	0.2892
k__Bacteria.p__Firmicutes.c__Clostridia.o__Clostridiales.f__Lachnospiraceae.g__Oribacterium	0.00041	0	0	0.2892
k__Bacteria.p__Firmicutes.c__Clostridia.o__Clostridiales.f__Peptococcaceae.g__Peptococcus	0.00022	0	0	0.2892
k__Bacteria.p__Firmicutes.c__Clostridia.o__Clostridiales.f__Peptostreptococcaceae.g__Filifactor	9.57E-06	0	0	0.2892
k__Bacteria.p__Firmicutes.c__Clostridia.o__Clostridiales.f__Peptostreptococcaceae.g__Peptostreptococcus	0.00067	0	0	0.2892
k__Bacteria.p__Firmicutes.c__Clostridia.o__Clostridiales.f__Ruminococcaceae.g__Faecalibacterium	0	4.8E-05	0	0.2892
k__Bacteria.p__Firmicutes.c__Clostridia.o__Clostridiales.f__Veillonellaceae.Other	9.57E-06	0	0	0.2892
k__Bacteria.p__Firmicutes.c__Clostridia.o__Clostridiales.f__Veillonellaceae.g__Megasphaera	0.00203	0	0	0.2892

k__Bacteria.p__Firmicutes.c__Clostridia.o__Clostridiales.f__Veillonellaceae.g__Selenomonas	0.00113	0	0	0.2892
k__Bacteria.p__Firmicutes.c__Clostridia.o__Clostridiales.f__.Mogibacteriaceae..g__	0.00063	0	0	0.2892
k__Bacteria.p__Firmicutes.c__Clostridia.o__Clostridiales.f__.Tissierellaceae..g__	7.7E-05	0	0	0.2892
k__Bacteria.p__Fusobacteria.c__Fusobacteriia.o__Fusobacteriales.f__Fusobacteriaceae.g__Cetobacterium	0	0.00011	0	0.2892
k__Bacteria.p__GN02.c__3BR.5F.o__.f__.g__	0	9.57E-06	0	0.2892
k__Bacteria.p__Planctomycetes.c__Planctomycetia.o__Gemmatales.f__Gemmataceae.g__Gemmata	0	0.00013	0	0.2892
k__Bacteria.p__Planctomycetes.c__Planctomycetia.o__Gemmatales.f__Isosphaeraceae.g__	0	0.0002	0	0.2892
k__Bacteria.p__Proteobacteria.c__Alphaproteobacteria.o__Ellin329.f__.g__	0	9.57E-06	0	0.2892
k__Bacteria.p__Proteobacteria.c__Alphaproteobacteria.o__Rhodobacterales.f__Rhodobacteraceae.g__Rhodobacter	0	7.7E-05	0	0.2892
k__Bacteria.p__Proteobacteria.c__Alphaproteobacteria.o__Rickettsiales.f__.g__	0	0.00039	0	0.2892
k__Bacteria.p__Proteobacteria.c__Alphaproteobacteria.o__Rickettsiales.f__Rickettsiaceae.g__	0	0.00023	0	0.2892
k__Bacteria.p__Proteobacteria.c__Alphaproteobacteria.o__Sphingomonadales.f__.g__	0	0.00032	0	0.2892
k__Bacteria.p__Proteobacteria.c__Betaproteobacteria.o__Burkholderiales.f__Alcaligenaceae.g__Sutterella	0	0.00011	0	0.2892
k__Bacteria.p__Proteobacteria.c__Betaproteobacteria.o__Burkholderiales.f__Comamonadaceae.g__Ramlibacter	0	9.57E-06	0	0.2892
k__Bacteria.p__Proteobacteria.c__Betaproteobacteria.o__Rhodocyclales.f__Rhodocyclaceae.g__Propionivibrio	9.57E-06	0	0	0.2892
k__Bacteria.p__Proteobacteria.c__Deltaproteobacteria.o__Myxococcales.f__Polyangiaceae.Other	6.7E-05	0	0	0.2892
k__Bacteria.p__Proteobacteria.c__Gammaproteobacteria.o__Xanthomonadales.f__Xanthomonadaceae.Other	2.9E-05	0	0	0.2892
k__Bacteria.p__Synergistetes.c__Synergistia.o__Synergistales.f__Dethiosulfovibrionaceae.g__TG5	1.91E-05	0	0	0.2892
k__Bacteria.p__TM7.c__.o__.f__.g__	0.00011	0	0	0.2892
k__Bacteria.p__TM7.c__TM7.3.o__CW040.f__.g__	0.00055	0	0	0.2892
k__Bacteria.p__Verrucomicrobia.c__Opitutae.o__Opitutales.f__Opitutaceae.g__Opitutus	9.57E-06	0	0	0.2892
k__Bacteria.p__Verrucomicrobia.c__.Spartobacteria..o__.Chthoniobacterales..f__.Chthoniobacteraceae..g__	0	0.00027	0	0.2892
k__Bacteria.p__Verrucomicrobia.c__.Spartobacteria..o__.Chthoniobacterales..f__.Chthoniobacteraceae..g__Candidatus.Xiphinematobacter	0	0.00036	0	0.2892
k__Bacteria.p__ZB3.c__BS119.o__.f__.g__	0	0.00015	0	0.2892
k__Bacteria.p__Cyanobacteria.c__Chloroplast.o__Streptophyta.f__.g__	0.00602	0.0263	0.03198	0.29009
k__Bacteria.p__Actinobacteria.c__Actinobacteria.o__Actinomycetales.f__Nocardiaceae.g__Rhodococcus	0	0	8.6E-05	0.29148
k__Bacteria.p__Verrucomicrobia.c__Opitutae.o__HA64.f__.g__	0	0	0.00014	0.29276
k__Bacteria.p__Firmicutes.c__Bacilli.o__Lactobacillales.f__.g__	0.0002	0.00126	0.00055	0.29354
k__Bacteria.p__Proteobacteria.c__Alphaproteobacteria.o__Rhizobiales.f__.g__	0	0.0004	5.7E-06	0.29491
k__Bacteria.p__Firmicutes.c__Erysipelotrichi.o__Erysipelotrichales.f__Erysipelotrichaceae.g__Bulleidia	0.00039	0	5.7E-06	0.29505
k__Bacteria.p__Cyanobacteria.c__4C0d.2.o__YS2.f__.g__	0	0	0.00015	0.29551
k__Bacteria.p__Proteobacteria.c__Betaproteobacteria.o__Burkholderiales.f__Comamonadaceae.g__Delftia	0.00032	0.00019	4.59E-05	0.29572
k__Bacteria.p__Actinobacteria.c__Actinobacteria.o__Actinomycetales.f__Nocardioidaceae.g__Aeromicrobium	9.6E-05	0.00072	0	0.29659
k__Bacteria.p__Proteobacteria.c__Gammaproteobacteria.o__Legionellales.f__Legionellaceae.g__	0	0.00045	4.59E-05	0.29857
k__Bacteria.p__Actinobacteria.c__Actinobacteria.o__Actinomycetales.f__Actinomycetaceae.Other	0	0.00044	1.1E-05	0.29948
k__Bacteria.p__Proteobacteria.c__Alphaproteobacteria.o__Rhodospirillales.f__Rhodospirillaceae.g__Azospirillum	0	0	8.04E-05	0.29958
k__Bacteria.p__Acidobacteria.c__.Chloracidobacteria..o__RB41.f__Ellin6075.g__	9.57E-06	0.00016	0	0.30317
k__Bacteria.p__Proteobacteria.c__Alphaproteobacteria.o__Sphingomonadales.f__Sphingomonadaceae.g__Sphingobium	0	0	0.00063	0.30456
k__Bacteria.p__Actinobacteria.c__Actinobacteria.o__Actinomycetales.f__Micrococcaceae.g__Renibacterium	0.00013	9.57E-06	0	0.30625
k__Bacteria.p__Actinobacteria.c__Rubrobacteria.o__Rubrobacterales.f__Rubrobacteraceae.g__Rubrobacter	0.00036	0	1.7E-05	0.30962
k__Bacteria.p__Proteobacteria.c__Alphaproteobacteria.o__.f__.g__	0.0004	3.8E-05	0	0.31211
k__Bacteria.p__Actinobacteria.c__Actinobacteria.o__Actinomycetales.f__Micrococcaceae.g__Kocuria	9.57E-06	0	0.00053	0.31422
k__Bacteria.p__Proteobacteria.c__Gammaproteobacteria.o__Legionellales.f__Legionellaceae.g__Legionella	0	8.6E-05	5.7E-06	0.31915
k__Bacteria.p__Firmicutes.c__Clostridia.o__Clostridiales.f__Clostridiaceae.g__Clostridium	0.00273	0.00136	0.00161	0.32116
k__Bacteria.p__Actinobacteria.c__Actinobacteria.o__Actinomycetales.f__Dermacoccaceae.g__Dermacoccus	0.00066	0.00019	0.00018	0.32335
k__Bacteria.p__Firmicutes.c__Clostridia.o__Clostridiales.f__Veillonellaceae.g__Veillonella	0.02822	0.00474	0.00191	0.32897
k__Bacteria.p__Proteobacteria.c__Alphaproteobacteria.o__Sphingomonadales.f__Erythrobacteraceae.g__	0	6.7E-05	5.7E-06	0.32921
k__Bacteria.p__Cyanobacteria.c__ML635J.21.o__.f__.g__	0.00046	0	4.02E-05	0.33019
k__Bacteria.p__Proteobacteria.c__Betaproteobacteria.o__Burkholderiales.f__Oxalobacteraceae.g__Janthinobacterium	0	9.57E-06	0.00086	0.33168
k__Bacteria.p__Bacteroidetes.c__Bacteroidia.o__Bacteroidales.f__Prevotellaceae.g__Prevotella	0.02799	0.00159	0.00454	0.33181
k__Bacteria.p__Firmicutes.c__Bacilli.o__Gemellales.f__Gemellaceae.g__Gemella	0.00521	0.04544	0.05972	0.33343
k__Bacteria.p__Firmicutes.c__Bacilli.o__Lactobacillales.f__Streptococcaceae.g__	5.74E-05	0.00014	5.7E-06	0.33362
k__Bacteria.p__Proteobacteria.c__Betaproteobacteria.o__Burkholderiales.f__Comamonadaceae.g__	0.00012	0.00124	0.00051	0.33768
k__Bacteria.p__Proteobacteria.c__Gammaproteobacteria.o__Enterobacteriales.f__Enterobacteriaceae.g__Erwinia	2.9E-05	1.91E-05	0.0007	0.33859
k__Bacteria.p__Proteobacteria.c__Betaproteobacteria.o__Methylophilales.f__Methylophilaceae.g__	1.91E-05	0.0002	1.1E-05	0.33964
k__Bacteria.p__Bacteroidetes.c__Flavobacteriia.o__Flavobacteriales.f__Flavobacteriaceae.g__Flavobacterium	4.8E-05	0.00035	5.17E-05	0.34026
k__Bacteria.p__Proteobacteria.c__Deltaproteobacteria.o__Desulfovibrionales.f__Desulfovibrionaceae.g__	0	0.00042	5.17E-05	0.34084
k__Bacteria.p__Firmicutes.c__Bacilli.o__Lactobacillales.f__Leuconostocaceae.g__Weissella	0	0	3.44E-05	0.34288
k__Bacteria.p__Proteobacteria.c__Gammaproteobacteria.o__Pseudomonadales.f__Moraxellaceae.g__Enhydrobacter	0.002	0.00036	0.00157	0.34575
k__Bacteria.p__Firmicutes.c__Clostridia.o__Clostridiales.f__Lachnospiraceae.g__Butyrivibrio	4.8E-05	0	5.7E-06	0.34886
k__Bacteria.p__Proteobacteria.c__Betaproteobacteria.o__Burkholderiales.f__Oxalobacteraceae.g__Polynucleobacter	0	9.6E-05	1.1E-05	0.34886
k__Bacteria.p__Actinobacteria.c__Actinobacteria.o__Actinomycetales.f__Dietziaceae.Other	0	0.00037	5.17E-05	0.34919
k__Bacteria.p__Deferribacteres.c__Deferribacteres.o__Deferribacterales.f__Deferribacteraceae.g__Mucispirillum	0	0	0.00032	0.35307
k__Bacteria.p__Proteobacteria.c__Alphaproteobacteria.o__Rhizobiales.f__Rhizobiaceae.g__Agrobacterium	0.00058	0.00104	0.00135	0.35625
k__Bacteria.p__Proteobacteria.c__Alphaproteobacteria.o__Rhizobiales.f__Hyphomicrobiaceae.g__Devosia	0	8.6E-05	1.1E-05	0.35699
k__Bacteria.p__Proteobacteria.c__Alphaproteobacteria.o__Rhodospirillales.f__Acetobacteraceae.g__	0	2.9E-05	1.1E-05	0.35699
k__Bacteria.p__Proteobacteria.c__Betaproteobacteria.o__Burkholderiales.f__Comamonadaceae.g__Comamonas	0	0	0.00119	0.36243
k__Bacteria.p__Actinobacteria.c__Actinobacteria.o__Actinomycetales.f__Bogoriellaceae.g__Georgenia	2.9E-05	3.83E-05	0.00012	0.36246
k__Bacteria.p__Proteobacteria.c__Betaproteobacteria.o__Neisseriales.f__Neisseriaceae.g__Neisseria	0.00401	6.7E-05	0.00083	0.3691
k__Bacteria.p__TM7.c__TM7.3.o__.f__.g__	0.00139	0	0.00028	0.37334
k__Bacteria.p__Proteobacteria.Other.Other.Other.Other	9.57E-06	6.7E-05	5.7E-06	0.37438
k__Bacteria.p__Proteobacteria.c__Gammaproteobacteria.o__Enterobacteriales.f__Enterobacteriaceae.g__Serratia	0.00013	4.8E-05	0	0.37575
k__Bacteria.p__Proteobacteria.c__Deltaproteobacteria.o__Desulfovibrionales.f__Desulfovibrionaceae.g__Desulfovibrio	0	0	0.00033	0.37799
k__Bacteria.p__Firmicutes.c__Clostridia.o__Clostridiales.f__Lachnospiraceae.g__.Ruminococcus.	4.8E-05	0.00065	0.0001	0.38082
k__Bacteria.p__Proteobacteria.c__Gammaproteobacteria.o__Pasteurellales.f__Pasteurellaceae.Other	3.8E-05	0	6.89E-05	0.38149
k__Bacteria.p__Actinobacteria.c__Actinobacteria.o__Actinomycetales.f__Actinomycetaceae.g__Actinomyces	0.00389	0.00209	0.00069	0.38492
k__Bacteria.p__Proteobacteria.c__Alphaproteobacteria.o__Caulobacterales.f__Caulobacteraceae.g__	0.00042	0.00034	0.00087	0.38534
k__Bacteria.p__Firmicutes.c__Bacilli.o__Gemellales.f__Gemellaceae.g__	0.0017	0.00286	0.00362	0.38795
k__Bacteria.p__Bacteroidetes.c__Bacteroidia.o__Bacteroidales.f__Rikenellaceae.g__	0	9.57E-06	0.00046	0.3914
k__Bacteria.p__Firmicutes.c__Bacilli.o__Gemellales.f__.g__	9.57E-06	0.00034	0.00057	0.40063
k__Bacteria.p__Proteobacteria.c__Gammaproteobacteria.o__Legionellales.f__.g__	0	5.7E-05	1.1E-05	0.40095
k__Bacteria.p__Proteobacteria.c__Gammaproteobacteria.Other.Other.Other	0	0	2.3E-05	0.40095
k__Bacteria.p__Proteobacteria.c__Gammaproteobacteria.o__Pasteurellales.f__Pasteurellaceae.g__	2.9E-05	0	5.7E-06	0.40095
k__Bacteria.p__Proteobacteria.c__Betaproteobacteria.o__Methylophilales.f__Methylophilaceae.g__Methylotenera	0	0	6.89E-05	0.40096
k__Bacteria.p__Bacteroidetes.c__Sphingobacteriia.o__Sphingobacteriales.f__Sphingobacteriaceae.g__	9.57E-06	1.91E-05	0	0.4037
k__Bacteria.p__Planctomycetes.c__Phycisphaerae.o__Phycisphaerales.f__.g__	1.91E-05	9.57E-06	0	0.4037
k__Bacteria.p__Bacteroidetes.c__Bacteroidia.o__Bacteroidales.f__Porphyromonadaceae.g__	0	0	0.00151	0.40662
k__Bacteria.p__Firmicutes.c__Clostridia.o__Clostridiales.f__.Tissierellaceae..g__Helcococcus	0.013	0.01404	0.00876	0.41212
k__Bacteria.p__Proteobacteria.c__Betaproteobacteria.o__Burkholderiales.f__Oxalobacteraceae.g__Ralstonia	8.6E-05	6.7E-05	0.00033	0.41857
k__Bacteria.p__Proteobacteria.c__Alphaproteobacteria.o__Rhizobiales.f__Phyllobacteriaceae.g__	0.00218	0.00164	0.00137	0.41942
k__Bacteria.p__Fusobacteria.c__Fusobacteriia.o__Fusobacteriales.f__Leptotrichiaceae.g__Leptotrichia	0.00098	0	0.00022	0.42161
k__Bacteria.p__Actinobacteria.c__Actinobacteria.o__Bifidobacteriales.f__Bifidobacteriaceae.g__Bifidobacterium	7.7E-05	0.00011	0	0.42604

k__Bacteria.p__Bacteroidetes.c__Bacteroidia.o__Bacteroidales.f__.Odoribacteraceae..g__Odoribacter	4.8E-05	0	1.1E-05	0.42915
k__Bacteria.p__Bacteroidetes.c__Bacteroidia.o__Bacteroidales.f__S24.7.g__	0.00111	0.00075	0.00201	0.42928
k__Bacteria.p__Firmicutes.c__Bacilli.o__Gemellales.Other.Other	9.57E-06	4.78E-05	2.9E-05	0.42946
k__Bacteria.p__Proteobacteria.c__Gammaproteobacteria.o__Enterobacteriales.f__Enterobacteriaceae.Other	0.0005	0.00139	0.00109	0.43014
k__Bacteria.p__Bacteroidetes.c__Bacteroidia.o__Bacteroidales.f__Porphyromonadaceae.g__Porphyromonas	0.00036	0.00011	3.44E-05	0.43517
k__Bacteria.p__Proteobacteria.c__Deltaproteobacteria.o__Bdellovibrionales.f__Bdellovibrionaceae.g__Bdellovibrio	0	0.00011	2.9E-05	0.43632
k__Bacteria.p__Firmicutes.c__Clostridia.o__Clostridiales.f__.Tissierellaceae..g__Parvimonas	0.00021	0.00025	0	0.4381
k__Bacteria.p__Firmicutes.c__Erysipelotrichi.o__Erysipelotrichales.f__Erysipelotrichaceae.g__	0	0	0.00014	0.44079
k__Bacteria.p__Actinobacteria.c__Coriobacteriia.o__Coriobacteriales.f__Coriobacteriaceae.g__Adlercreutzia	0.00019	0	0.00023	0.44314
k__Bacteria.p__Firmicutes.c__Bacilli.o__Lactobacillales.f__Enterococcaceae.g__	0.00022	0.00012	5.2E-05	0.44435
k__Bacteria.p__Proteobacteria.c__Alphaproteobacteria.o__Rickettsiales.f__mitochondria.Other	0.00448	0.01322	0.01467	0.4464
k__Bacteria.p__Bacteroidetes.c__Flavobacteriia.o__Flavobacteriales.f__.Weeksellaceae..g__Cloacibacterium	0	9.57E-06	0.00019	0.4476
k__Bacteria.p__Proteobacteria.c__Alphaproteobacteria.o__Rhizobiales.f__Phyllobacteriaceae.g__Aminobacter	0	1.91E-05	1.7E-05	0.44761
k__Bacteria.p__TM6.c__SJA.4.o__.f__.g__	0	0	0.00026	0.45312
k__Bacteria.p__Proteobacteria.c__Betaproteobacteria.o__Rhodocyclales.f__Rhodocyclaceae.Other	0.00011	9.57E-06	2.30E-05	0.45322
k__Bacteria.p__Actinobacteria.c__Actinobacteria.o__Actinomycetales.f__Actinomycetaceae.g__Arcanobacterium	0.02861	0.03215	0.02287	0.45508
k__Bacteria.p__Proteobacteria.c__Gammaproteobacteria.o__Pasteurellales.f__Pasteurellaceae.g__Haemophilus	0.00281	0.00037	0.00105	0.45696
k__Bacteria.p__Proteobacteria.c__Gammaproteobacteria.o__Enterobacteriales.f__Enterobacteriaceae.g__Trabulsiella	2.9E-05	7.65E-05	4.02E-05	0.46045
k__Bacteria.p__Bacteroidetes.c__Sphingobacteriia.o__Sphingobacteriales.f__Sphingobacteriaceae.g__Pedobacter	0	0	0.00045	0.46203
k__Bacteria.p__Proteobacteria.c__Alphaproteobacteria.o__Rhizobiales.f__Brucellaceae.g__Ochrobactrum	0.00034	0.00066	0.00028	0.46422
k__Bacteria.p__Proteobacteria.c__Betaproteobacteria.o__Burkholderiales.f__Comamonadaceae.Other	0	0	3.4E-05	0.46822
k__Bacteria.p__Firmicutes.c__Bacilli.o__Bacillales.f__Bacillaceae.g__Anaerobacillus	0	1.91E-05	5.7E-06	0.47227
k__Bacteria.p__Proteobacteria.c__Gammaproteobacteria.o__Enterobacteriales.f__Enterobacteriaceae.g__Enterobacter	1.91E-05	0	5.7E-06	0.47227
k__Bacteria.p__Actinobacteria.c__Actinobacteria.o__Actinomycetales.f__Micrococcaceae.g__	0.00608	0.00809	0.00925	0.48016
k__Bacteria.p__Firmicutes.c__Bacilli.o__Bacillales.f__Bacillaceae.g__	0.00011	0.00077	0.00047	0.49121
k__Bacteria.p__Firmicutes.c__Bacilli.o__Lactobacillales.f__Aerococcaceae.Other	0	0	0.0004	0.49964
k__Bacteria.p__Firmicutes.c__Bacilli.o__Bacillales.f__Planococcaceae.g__Sporosarcina	0.00044	0	0.00015	0.49997
k__Bacteria.p__Proteobacteria.c__Alphaproteobacteria.o__Rhizobiales.f__Methylobacteriaceae.g__	0	0	0.00045	0.50127
k__Bacteria.p__Fusobacteria.c__Fusobacteriia.o__Fusobacteriales.f__Fusobacteriaceae.g__Fusobacterium	0.00126	0	0.00051	0.50665
k__Bacteria.p__Firmicutes.c__Clostridia.o__Clostridiales.f__.g__	0.00264	0.00634	0.0068	0.52194
k__Bacteria.p__Proteobacteria.c__Gammaproteobacteria.o__Alteromonadales.f__Shewanellaceae.g__Shewanella	0.00044	4.8E-05	0.0002	0.52602
k__Bacteria.p__Actinobacteria.c__Actinobacteria.o__Actinomycetales.f__Microbacteriaceae.g__Pseudoclavibacter	0.00267	0.00136	0.00242	0.53026
k__Bacteria.p__Actinobacteria.c__Actinobacteria.o__Actinomycetales.f__Micrococcaceae.g__Rothia	0.00276	0.00056	0.00121	0.54166
k__Bacteria.p__Firmicutes.c__Bacilli.o__Bacillales.f__Bacillaceae.g__Bacillus	0.00057	6.70E-05	0.00039	0.54293
k__Bacteria.p__Bacteroidetes.c__.Saprospirae..o__.Saprospirales..f__Chitinophagaceae.g__Flavisolibacter	3.8E-05	0	0.00042	0.54371
k__Bacteria.p__Spirochaetes.c__Spirochaetes.o__Spirochaetales.f__Spirochaetaceae.g__Treponema	0.00059	0	0.00024	0.54514
k__Bacteria.p__Proteobacteria.c__Alphaproteobacteria.o__Rhodospirillales.f__Acetobacteraceae.g__Acetobacter	0.00144	0.00123	0.0007	0.55039
k__Bacteria.p__Firmicutes.c__Clostridia.o__Clostridiales.f__.Mogibacteriaceae..g__Anaerovorax	0	0	0.00026	0.55061
k__Bacteria.p__Firmicutes.c__Clostridia.o__Clostridiales.f__Veillonellaceae.g__Dialister	0	0.00045	0.00019	0.55449
k__Bacteria.p__Proteobacteria.c__Alphaproteobacteria.o__Rhizobiales.f__Rhizobiaceae.Other	0	0	0.00011	0.56243
k__Bacteria.p__Firmicutes.c__Bacilli.o__Lactobacillales.f__Aerococcaceae.g__Facklamia	0.0029	0.00413	0.0017	0.56891
k__Bacteria.p__Firmicutes.c__Clostridia.o__Clostridiales.f__Ruminococcaceae.g__Oscillospira	0.0002	0.00112	0.00145	0.57637
k__Bacteria.p__Actinobacteria.c__Actinobacteria.o__Actinomycetales.f__Micrococcaceae.g__Arthrobacter	9.57E-06	0.00011	0.00042	0.57831
k__Bacteria.p__Firmicutes.c__Bacilli.o__Bacillales.f__Staphylococcaceae.Other	2.9E-05	9.57E-06	1.1E-05	0.57838
k__Bacteria.p__Firmicutes.c__Clostridia.o__Clostridiales.f__Peptostreptococcaceae.g__	0	0	0.00025	0.58267
k__Bacteria.p__Proteobacteria.c__Gammaproteobacteria.o__Pseudomonadales.f__Pseudomonadaceae.g__Pseudomonas	0.01004	0.00351	0.00491	0.58696
k__Bacteria.p__Proteobacteria.c__Gammaproteobacteria.o__Pseudomonadales.f__Moraxellaceae.g__Psychrobacter	0	9.57E-06	0.00021	0.59287
k__Bacteria.p__Firmicutes.c__Bacilli.o__Lactobacillales.f__Aerococcaceae.g__Aerococcus	0.00027	0	0.00013	0.59592
k__Bacteria.p__Acidobacteria.c__Acidobacteria.6.o__iii1.15.f__.g__	0	0	0.00013	0.5997
k__Bacteria.p__Actinobacteria.c__Actinobacteria.o__Actinomycetales.f__Dermabacteraceae.g__Brachybacterium	0	0	0.0002	0.5997
k__Bacteria.p__Actinobacteria.c__Actinobacteria.o__Actinomycetales.f__Intrasporangiaceae.g__Janibacter	0	0	0.00018	0.5997
k__Bacteria.p__Actinobacteria.c__Actinobacteria.o__Actinomycetales.f__Microbacteriaceae.g__	0	0	5.17E-05	0.5997
k__Bacteria.p__Actinobacteria.c__Actinobacteria.o__Actinomycetales.f__Micrococcaceae.g__Nesterenkonia	0	0	5.7E-06	0.5997
k__Bacteria.p__Actinobacteria.c__Actinobacteria.o__Actinomycetales.f__Nocardioidaceae.g__Nocardioides	0	0	3.44E-05	0.5997
k__Bacteria.p__Actinobacteria.c__Thermoleophilia.o__Solirubrobacterales.Other.Other	0	0	4.59E-05	0.5997
k__Bacteria.p__Armatimonadetes.c__Armatimonadia.o__Armatimonadales.f__Armatimonadaceae.g__	0	0	0.00013	0.5997
k__Bacteria.p__Bacteroidetes.c__Bacteroidia.o__Bacteroidales.f__Rikenellaceae.g__AF12	0	0	0.00021	0.5997
k__Bacteria.p__Bacteroidetes.c__Cytophagia.o__Cytophagales.f__Cytophagaceae.g__	0	0	5.17E-05	0.5997
k__Bacteria.p__Bacteroidetes.c__Cytophagia.o__Cytophagales.f__Cytophagaceae.g__Hymenobacter	0	0	5.7E-05	0.5997
k__Bacteria.p__Bacteroidetes.c__.Rhodothermi..o__.Rhodothermales..f__.Balneolaceae..g__KSA1	0	0	0.00018	0.5997
k__Bacteria.p__Chlamydiae.c__Chlamydiia.o__Chlamydiales.f__.g__	0	0	5.7E-05	0.5997
k__Bacteria.p__Chlamydiae.c__Chlamydiia.o__Chlamydiales.f__Parachlamydiaceae.Other	0	0	5.7E-06	0.5997
k__Bacteria.p__Cyanobacteria.c__Oscillatoriophycideae.o__Chroococcales.f__Xenococcaceae.g__	0	0	6.89E-05	0.5997
k__Bacteria.p__Firmicutes.c__Bacilli.o__Bacillales.f__Planococcaceae.g__Planomicrobium	0	0	5.7E-06	0.5997
k__Bacteria.p__Firmicutes.c__Bacilli.o__Bacillales.f__.Exiguobacteraceae..g__	0	0	1.1E-05	0.5997
k__Bacteria.p__Firmicutes.c__Bacilli.o__Lactobacillales.f__Carnobacteriaceae.g__	0	0	0.00013	0.5997
k__Bacteria.p__Firmicutes.c__Bacilli.o__Lactobacillales.f__Enterococcaceae.g__Melissococcus	0	0	1.1E-05	0.5997
k__Bacteria.p__Firmicutes.c__Bacilli.o__Lactobacillales.f__Lactobacillaceae.g__	0	0	0.00025	0.5997
k__Bacteria.p__Firmicutes.c__Bacilli.o__Lactobacillales.f__Streptococcaceae.Other	0	0	5.7E-06	0.5997
k__Bacteria.p__Firmicutes.c__Clostridia.o__Clostridiales.f__Christensenellaceae.g__	0	0	1.1E-05	0.5997
k__Bacteria.p__Firmicutes.c__Clostridia.o__Clostridiales.f__Clostridiaceae.g__	0	0	1.7E-05	0.5997
k__Bacteria.p__Firmicutes.c__Clostridia.o__Clostridiales.f__Eubacteriaceae.g__Acetobacterium	0	0	1.1E-05	0.5997
k__Bacteria.p__Firmicutes.c__Clostridia.o__Clostridiales.f__Lachnospiraceae.Other	0	0	1.7E-05	0.5997
k__Bacteria.p__Firmicutes.c__Clostridia.o__Clostridiales.f__Peptococcaceae.g__	0	0	1.1E-05	0.5997
k__Bacteria.p__Firmicutes.c__Clostridia.o__Clostridiales.f__Veillonellaceae.g__Anaeromusa	0	0	0.0004	0.5997
k__Bacteria.p__Gemmatimonadetes.c__Gemmatimonadetes.o__.f__.g__	0	0	5.17E-05	0.5997
k__Bacteria.p__OD1.c__.o__.f__.g__	0	0	7.46E-05	0.5997
k__Bacteria.p__OD1.c__SM2F11.o__.f__.g__	0	0	8.6E-05	0.5997
k__Bacteria.p__Planctomycetes.c__Planctomycetia.o__Pirellulales.f__Pirellulaceae.g__	0	0	2.9E-05	0.5997
k__Bacteria.p__Proteobacteria.c__Alphaproteobacteria.o__Caulobacterales.f__Caulobacteraceae.g__Brevundimonas	0	0	0.0001	0.5997
k__Bacteria.p__Proteobacteria.c__Alphaproteobacteria.o__Rhodobacterales.f__Rhodobacteraceae.g__	0	0	0.00017	0.5997
k__Bacteria.p__Proteobacteria.c__Alphaproteobacteria.o__Rhodospirillales.f__Acetobacteraceae.g__Gluconobacter	0	0	0.0002	0.5997
k__Bacteria.p__Proteobacteria.c__Alphaproteobacteria.o__Rhodospirillales.f__Rhodospirillaceae.g__	0	0	0.00025	0.5997
k__Bacteria.p__Proteobacteria.c__Alphaproteobacteria.o__Sphingomonadales.f__Sphingomonadaceae.g__Sphingopyxis	0	0	5.7E-06	0.5997
k__Bacteria.p__Proteobacteria.c__Betaproteobacteria.Other.Other.Other	0	0	1.1E-05	0.5997
k__Bacteria.p__Proteobacteria.c__Betaproteobacteria.o__Burkholderiales.f__Comamonadaceae.g__Curvibacter	0	0	8.6E-05	0.5997
k__Bacteria.p__Proteobacteria.c__Betaproteobacteria.o__Burkholderiales.f__Oxalobacteraceae.g__Cupriavidus	0	0	1.1E-05	0.5997
k__Bacteria.p__Proteobacteria.c__Deltaproteobacteria.o__MIZ46.f__.g__	0	0	1.7E-05	0.5997
k__Bacteria.p__Proteobacteria.c__Gammaproteobacteria.o__Cardiobacteriales.f__Cardiobacteriaceae.g__Cardiobacterium	0	0	0.00015	0.5997
k__Bacteria.p__Proteobacteria.c__Gammaproteobacteria.o__Chromatiales.f__.g__	0	0	0.00021	0.5997
k__Bacteria.p__Proteobacteria.c__Gammaproteobacteria.o__Pseudomonadales.Other.Other	0	0	1.1E-05	0.5997
k__Bacteria.p__Proteobacteria.c__Gammaproteobacteria.o__Pseudomonadales.f__Moraxellaceae.g__	0	0	1.1E-05	0.5997

k__Bacteria.p__Proteobacteria.c__Gammaproteobacteria.o__Pseudomonadales.f__Pseudomonadaceae.Other	0	0	5.7E-06	0.5997
k__Bacteria.p__Proteobacteria.c__Gammaproteobacteria.o__Xanthomonadales.f__Xanthomonadaceae.g__Luteibacter	0	0	2.30E-05	0.5997
k__Bacteria.p__Proteobacteria.c__Gammaproteobacteria.o__Xanthomonadales.f__Xanthomonadaceae.g__Lysobacter	0	0	4.02E-05	0.5997
k__Bacteria.p__TM7.c__TM7.3.o__CW040.f__F16.g__	0	0	0.00025	0.5997
k__Bacteria.p__.Thermi..c__Deinococci.o__Deinococcales.f__Deinococcaceae.g__Deinococcus	0	0	7.46E-05	0.5997
k__Bacteria.p__Actinobacteria.c__Actinobacteria.o__Actinomycetales.f__Beutenbergiaceae.g__Salana	0	0	3.44E-05	0.5997
k__Bacteria.p__Armatimonadetes.c__Armatimonadia.o__Armatimonadales.f__Armatimonadaceae.g__Armatimonas	0	0	3.44E-05	0.5997
k__Bacteria.p__Chlamydiae.c__Chlamydiia.o__Chlamydiales.f__Rhabdochlamydiaceae.g__Candidatus.Rhabdochlamydia	0	0	3.44E-05	0.5997
k__Bacteria.p__Proteobacteria.c__Gammaproteobacteria.o__Salinisphaerales.f__Salinisphaeraceae.g__Salinisphaera	0	9.57E-06	0.00136	0.60172
k__Bacteria.p__Firmicutes.c__Clostridia.o__Clostridiales.f__Lachnospiraceae.g__	0.00105	0.00239	0.00126	0.61075
k__Bacteria.p__Firmicutes.c__Bacilli.o__Lactobacillales.f__Aerococcaceae.g__	0	0.00022	0.00011	0.61076
k__Bacteria.p__Firmicutes.c__Bacilli.o__Bacillales.f__Staphylococcaceae.g__Jeotgalicoccus	0	9.57E-06	0.00018	0.61451
k__Bacteria.p__Proteobacteria.c__Deltaproteobacteria.o__Myxococcales.f__.g__	0	9.57E-06	0.00013	0.62019
k__Bacteria.p__Firmicutes.c__Bacilli.o__Bacillales.f__Planococcaceae.g__	0.00383	0.0048	0.00358	0.6202
k__Bacteria.p__Proteobacteria.c__Alphaproteobacteria.o__Rhizobiales.f__Bradyrhizobiaceae.Other	0	5.7E-05	0.00011	0.62142
k__Bacteria.p__Bacteroidetes.c__Flavobacteriia.o__Flavobacteriales.f__Flavobacteriaceae.g__Capnocytophaga	0.00049	0	0.00073	0.62412
k__Bacteria.p__Actinobacteria.c__Actinobacteria.o__Actinomycetales.f__Gordoniaceae.g__Gordonia	1.91E-05	0.00016	0.00013	0.63502
k__Bacteria.p__Firmicutes.c__Bacilli.Other.Other.Other	0	9.57E-06	5.7E-06	0.64537
k__Bacteria.p__Proteobacteria.c__Betaproteobacteria.o__.f__.g__	0	0.00014	8.6E-05	0.64537
k__Bacteria.p__TM7.c__TM7.1.o__.f__.g__	2.9E-05	0	0.00015	0.65006
k__Bacteria.p__Proteobacteria.c__Alphaproteobacteria.o__Sphingomonadales.f__Sphingomonadaceae.Other	9.57E-06	0	4.59E-05	0.65579
k__Bacteria.p__Actinobacteria.c__Actinobacteria.o__Actinomycetales.f__Dietziaceae.g__Dietzia	0	4.8E-05	0.00022	0.65709
k__Bacteria.p__Firmicutes.c__Clostridia.o__Clostridiales.f__Clostridiaceae.Other	9.57E-06	2.9E-05	0.00019	0.65842
k__Bacteria.p__Actinobacteria.c__Actinobacteria.o__Actinomycetales.f__Nocardioidaceae.g__	0	1.91E-05	8.04E-05	0.66292
k__Bacteria.p__Firmicutes.c__Bacilli.o__Lactobacillales.f__Aerococcaceae.g__Alloiococcus	1.91E-05	0	7.46E-05	0.66717
k__Bacteria.p__SR1.c__.o__.f__.g__	5.7E-05	0	5.17E-05	0.66736
k__Bacteria.p__Proteobacteria.c__Gammaproteobacteria.o__Xanthomonadales.f__Xanthomonadaceae.g__	0.0002	0	0.00025	0.66771
k__Bacteria.p__Actinobacteria.c__Actinobacteria.o__Actinomycetales.f__Actinomycetaceae.g__Actinobaculum	0	4.8E-05	0.00017	0.6741
k__Bacteria.p__Cyanobacteria.c__4C0d.2.o__MLE1.12.f__.g__	0	4.8E-05	0.00015	0.68113
k__Bacteria.p__Proteobacteria.c__Gammaproteobacteria.o__Aeromonadales.f__Aeromonadaceae.g__	0.00081	0.00031	0.00038	0.6938
k__Bacteria.p__Firmicutes.c__Bacilli.o__Gemellales.f__Gemellaceae.Other	0	9.57E-06	2.30E-05	0.69912
k__Bacteria.p__Firmicutes.c__Bacilli.o__Lactobacillales.f__Enterococcaceae.g__Vagococcus	0.00015	9.57E-06	0.00015	0.70622
k__Bacteria.p__Planctomycetes.c__Planctomycetia.o__Gemmatales.f__Gemmataceae.g__	0	8.6E-05	7.46E-05	0.71157
k__Bacteria.p__Firmicutes.c__Bacilli.o__Bacillales.f__Planococcaceae.g__Rummeliibacillus	0.00012	0	0.00015	0.71357
k__Bacteria.p__Actinobacteria.c__Actinobacteria.o__Actinomycetales.f__Intrasporangiaceae.Other	0	1.91E-05	1.7E-05	0.71569
k__Bacteria.p__Proteobacteria.c__Alphaproteobacteria.o__Rhodospirillales.f__Acetobacteraceae.Other	9.57E-06	2.9E-05	1.1E-05	0.71569
k__Bacteria.p__Proteobacteria.c__Alphaproteobacteria.o__Sphingomonadales.f__Sphingomonadaceae.g__	7.7E-05	9.57E-06	0.00015	0.71782
k__Bacteria.p__Proteobacteria.c__Gammaproteobacteria.o__Enterobacteriales.f__Enterobacteriaceae.g__Klebsiella	0.00021	0.00068	0.00042	0.71813
k__Bacteria.p__Firmicutes.c__Clostridia.o__Clostridiales.Other.Other	0	9.57E-06	1.7E-05	0.71816
k__Bacteria.p__Firmicutes.c__Clostridia.o__Clostridiales.f__.Tissierellaceae..g__Anaerococcus	0.03449	0.0322	0.02871	0.72006
k__Bacteria.p__Proteobacteria.c__Deltaproteobacteria.o__Desulfovibrionales.f__Desulfovibrionaceae.Other	2.9E-05	0	4.59E-05	0.72446
k__Bacteria.p__Proteobacteria.c__Betaproteobacteria.o__Burkholderiales.f__Alcaligenaceae.g__Achromobacter	0	0.00011	0.00018	0.72446
k__Bacteria.p__Proteobacteria.c__Alphaproteobacteria.o__Rhodobacterales.f__Rhodobacteraceae.g__Amaricoccus	7.7E-05	0	8.04E-05	0.72722
k__Bacteria.p__Proteobacteria.c__Betaproteobacteria.o__Rhodocyclales.f__Rhodocyclaceae.g__Zoogloea	0.0002	0	0.00022	0.72866
k__Bacteria.p__Proteobacteria.c__Alphaproteobacteria.o__Rhodobacterales.f__Rhodobacteraceae.g__Paracoccus	0	0.0002	0.00026	0.73095
k__Bacteria.p__Actinobacteria.c__Actinobacteria.o__Actinomycetales.f__Microbacteriaceae.g__Microbacterium	0.00016	0.00032	0.00021	0.75145
k__Bacteria.p__Firmicutes.c__Bacilli.o__Bacillales.f__Bacillaceae.Other	7.65E-05	3.83E-05	0.00019	0.7577
k__Bacteria.p__Firmicutes.c__Bacilli.o__Lactobacillales.f__Enterococcaceae.Other	0.00116	0.00172	0.00098	0.76476
k__Bacteria.p__Firmicutes.c__Bacilli.o__Bacillales.Other.Other	0.00061	0.00037	0.0005	0.76886
k__Bacteria.p__Firmicutes.c__Bacilli.o__Lactobacillales.f__Lactobacillaceae.g__Lactobacillus	0.00108	0.00072	0.00062	0.76961
k__Bacteria.p__Firmicutes.c__Clostridia.o__Clostridiales.f__Ruminococcaceae.g__Ruminococcus	0.00011	0.00033	0.00037	0.77236
k__Bacteria.p__Proteobacteria.c__Betaproteobacteria.o__Burkholderiales.f__Burkholderiaceae.g__Lautropia	0.00018	2.9E-05	0.00017	0.79238
k__Bacteria.p__Firmicutes.c__Clostridia.o__Clostridiales.f__.Tissierellaceae..g__Peptoniphilus	0.00599	0.00718	0.00749	0.79388
k__Bacteria.p__Actinobacteria.c__Actinobacteria.o__Actinomycetales.Other.Other	0.00034	0.00077	0.00055	0.80237
k__Bacteria.p__Proteobacteria.c__Alphaproteobacteria.o__Rhizobiales.f__Bradyrhizobiaceae.g__	8.6E-05	3.8E-05	0.00014	0.84204
k__Bacteria.p__Firmicutes.c__Clostridia.o__Clostridiales.f__Lachnospiraceae.g__Moryella	0.00221	0.00162	0.00163	0.8713
k__Bacteria.p__Bacteroidetes.c__Sphingobacteriia.o__Sphingobacteriales.f__Sphingobacteriaceae.g__Sphingobacterium	0.00052	0.00094	0.0006	0.88634
k__Bacteria.p__Firmicutes.c__Clostridia.o__Clostridiales.f__.Tissierellaceae..g__Finegoldia	0.00685	0.00569	0.00666	0.91703
k__Bacteria.p__Bacteroidetes.c__Bacteroidia.o__Bacteroidales.f__Bacteroidaceae.g__Bacteroides	0.00154	0.00213	0.00154	0.92707
k__Bacteria.p__Proteobacteria.c__Alphaproteobacteria.o__Rhizobiales.f__Methylobacteriaceae.g__Methylobacterium	0.00033	0.00028	0.00024	0.9394
k__Bacteria.p__Actinobacteria.c__Actinobacteria.o__Actinomycetales.f__Propionibacteriaceae.g__Propionibacterium	0.00124	0.00126	0.00142	0.94828
k__Bacteria.p__Proteobacteria.c__Gammaproteobacteria.o__Xanthomonadales.f__Xanthomonadaceae.g__Stenotrophomonas	0.00205	0.00242	0.00219	0.96167
k__Bacteria.p__Proteobacteria.c__Alphaproteobacteria.o__Rhizobiales.f__Phyllobacteriaceae.g__Phyllobacterium	0.00022	0.00024	0.00028	0.9742
k__Bacteria.p__Bacteroidetes.c__Flavobacteriia.o__Flavobacteriales.f__.Weeksellaceae..g__Chryseobacterium	0.00079	0.00068	0.00086	0.97589

Supplementary Table S2. Primer sequences for DGGE, 16S Illumina high-throughput sequencing and qPCR-amplified genes.

Target Gene	Forward primer sequence (5’- 3’)	Reverse primer sequence (5’ - 3’)
***16S rRNA* gene (PCR - DGGE)**	(P3_GC-341F)<br>5’-CGC CCG CCG GGC GCG GCG GGC GGG GCG GGG GCA CGG GGG GCC TAC GGG AGG CAG CAG-3’	(P2_518R)<br>5’-ATT ACC GCG GCT GCT GG-3’
***16S rRNA* gene (PCR - Illumina)**	5’-TCG TCG GCA GCG TCA GAT GTC TAT AAG AGA CAG CCT ACG GGN GGC WGC AG-3’	5’-GTC TCG TGG GCT CGG AGA TGT GTA TAA GAG ACA GGA CTA CHV GGG TAT CTA ATC C-3’
Eubacterial Universal 16S	5’-ACT CCT ACG GGA GGC AGC AGT-3’	5’-ATT ACC GCG GCTGCT GGC-3’
P. aeruginosa	5’-TTC CCT TGA TCA GGT CGA TCT-3’	5’-CCT GGT TGA TCA GGT CGA TCT-3’
P. acnes	5’-ATA CGT AGG GTG CGA GCG TTG TCC-3’	5’-TGG TGT TCC TCC TGA TAT CTG CGC-3’
S. aureus	5’-GCG ATT GAT GGT GAT ACG GTT-3’	5’-AGC CAA GCC TTG ACG AAC TAA-3’
S. epidermidis	5’-TCA GCA GTT GAA GGG ACA GAT-3’	5’-CCA GAA CAA TGA ATG GTT AAG G-3’
mBD-1	5’-TTT CAC ATC CTC TCT GCA CT-3’	5’-ACC TGG CTC CAT CTG GGA GA-3’
mBD-3	5’-CTC TTT GCA TTT CTC CTG GTG CTG CTG-3’	5’-CAT CTT CAT GGA GGA GCA AAT TCT G-3’
mBD-14	5’-ATC CAG ATC TGG GTA CCG ACG ACG ACG ACA ACT TCC TAC CAA AAA CCC TCC-3’	5’-ATT TGC GGG CGC CTA CTT CTT CTT TCG GCA GC-3’
mIL-22	5’-AGC TTG AGG TGT CCA ACT TC-3’	5’-GGT AGC ACT CAT CCT TAG CAC TG-3’
